# Acetylated histone variant H2A.Z is involved in the activation of neo-enhancers in prostate cancer

**DOI:** 10.1038/s41467-017-01393-8

**Published:** 2017-11-07

**Authors:** Fátima Valdés-Mora, Cathryn M. Gould, Yolanda Colino-Sanguino, Wenjia Qu, Jenny Z. Song, Kylie M. Taylor, Fabian A. Buske, Aaron L. Statham, Shalima S. Nair, Nicola J. Armstrong, James G. Kench, Kenneth M. L. Lee, Lisa G. Horvath, Minru Qiu, Alexei Ilinykh, Nicole S. Yeo-Teh, David Gallego-Ortega, Clare Stirzaker, Susan J. Clark

**Affiliations:** 1 Histone Variants Group, Genomics and Epigenetics Division, Garvan Institute of Medical Research, Sydney, NSW 2010 Australia; 2 Epigenetics Research Laboratory, Genomics and Epigenetics Division, Garvan Institute of Medical Research, Sydney, NSW 2010 Australia; 3 St. Vincent’s Clinical School, UNSW Sydney, Sydney, NSW 2010 Australia; 4 Mathematics and Statistics, Murdoch University, Perth, WA 6150 Australia; 5 Department of Tissue Pathology and Diagnostic Oncology, Royal Prince Alfred Hospital, Camperdown, NSW 2050 Australia; 6 Sydney Medical School, University of Sydney, Sydney, NSW 2050 Australia; 7 Clinical Prostate Cancer Research Group, Cancer Division, Garvan Institute of Medical Research, Sydney, NSW 2010 Australia; 8 Anatomical Pathology Department, Concord Hospital, Sydney, NSW 2139 Australia; 9 Chris O’Brien Lifehouse, Sydney, NSW 2050 Australia; 10 Department of Anatomical Pathology, Sydpath, St Vincent’s Hospital, Sydney, NSW 2010 Australia; 11 Tumour Development Group. Cancer Division, Garvan Institute of Medical Research, Sydney, NSW 2010 Australia

## Abstract

Acetylation of the histone variant H2A.Z (H2A.Zac) occurs at active promoters and is associated with oncogene activation in prostate cancer, but its role in enhancer function is still poorly understood. Here we show that H2A.Zac containing nucleosomes are commonly redistributed to neo-enhancers in cancer resulting in a concomitant gain of chromatin accessibility and ectopic gene expression. Notably incorporation of acetylated H2A.Z nucleosomes is a pre-requisite for activation of Androgen receptor (AR) associated enhancers. H2A.Zac nucleosome occupancy is rapidly remodeled to flank the AR sites to initiate the formation of nucleosome-free regions and the production of AR-enhancer RNAs upon androgen treatment. Remarkably higher levels of global H2A.Zac correlate with poorer prognosis. Altogether these data demonstrate the novel contribution of H2A.Zac in activation of newly formed enhancers in prostate cancer.

## Abstract (web summary)

Acetylation of the histone variant H2A.Z at gene promoters is associated with oncogene activation; however, it is unclear if such modification has a role in regulating the function of enhancers. Here the authors show that acetylated H2A.Z is redistributed at cancer neo-enhancers and regulates the activity of specific enhancers of cancer-related genes.

## Introduction

Histone variants have evolved to carry out functions that are distinct from those of the major core histones^1^. H2A.Z is an evolutionary conserved variant of the canonical H2A, which shares only 60% sequence identity. H2A.Z nucleosome occupancy is associated with a wide variety of opposing functions^2^, including regulation of gene transcription. H2A.Z has been implicated in active, poised and inactive gene expression. One of the main mechanisms to explain these contrasting roles of H2A.Z is post-translation modifications. Mammalian H2A.Z can be modified by acetylation, monoubiquitylation or methylation at specific lysines^3^. In particular the acetylated form(s) of H2A.Z (K4, K7, and K11; H2A.Zac) are reported to be associated with active chromatin at promoter regions^4, 5^ and confer nucleosome destabilization and open conformation^6^.

An oncogenic role for H2A.Z has previously been suggested^7^. H2A.Z expression is increased in several human malignancies including glioma, melanoma, colorectal, breast, bladder and lung cancer (Reviewed in ref. ^7^). Recently, we^5^ and others^8, 9^ suggested a role for H2A.Z in deregulation of gene expression and tumor progression in prostate cancer. We found that acetylation of H2A.Z is associated with gene mis-expression in prostate cancer and suggested a model whereby oncogenes are activated after gaining acetylation at H2A.Z-nucleosomes at gene promoters and conversely tumor suppressor genes are silenced concomitant with the loss of H2A.Zac at promoters^5^.

The histone variant H2A.Z and its acetylation status therefore appears to play a key role in tumourigenesis. However previous studies have not addressed the genome-wide distribution of acetylated H2A.Z beyond gene promoters and the associated molecular consequences in cancer. H2A.Z has been reported to mark gene enhancers in hematopoietic cells^10^, ES cells^11, 12^, and HeLa cells^13^. However the genome-wide profile of H2A.Zac has only been studied in ES cells and was associated with active enhancers^11, 12^. Although the combination of H3K4Me1, H3K27ac, and H2A.Z are highly predictive of enhancer position and activity^13, 14^, direct evidence supporting functionality of enhancer chromatin modifications is still lacking. A recent publication showed that H2A.Z is essential for estrogen receptor (ER) enhancer activity, as it is required for RNA polymerase II (RNA Pol II) recruitment and enhancer RNAs (eRNAs) transcription^15^. However insights into the role of the acetylated form of H2A.Z in normal or cancer-related enhancer functions are still lacking.

A well-known model to study epigenetic mechanisms of enhancer regulation in prostate cancer is the transcriptional regulation mediated by the ligand activated transcription factor, androgen receptor (AR)^16, 17^. AR is a key regulator of prostate growth and prostate cancer progression through the regulation of AR target genes^17^. AR binds to the DNA at specific regions (AREs) mainly located at distal regions of AR-regulated genes^17^. At least some of these AR distal regions are important for transcriptional regulation, and termed as “AR-enhancers”, characterized by the presence of RNA Pol II, eRNAs, H3K4Me1, H3K27ac, nucleosome depleted regions (NDRs), and H2A.Z^16, 18^. However the involvement of H2A.Z acetylation during AR signaling in prostate cancer has not been studied yet. Here, we provide the first comprehensive study characterising the global dynamics and function of H2A.Zac at enhancers in prostate cancer and demonstrate that H2A.Zac plays an active role in AR-enhancer function during androgen treatment suggesting an pro-oncogenic role in cancer.

## Results

### H2A.Zac clinical relevance in prostate cancer

**Fig. 1:**
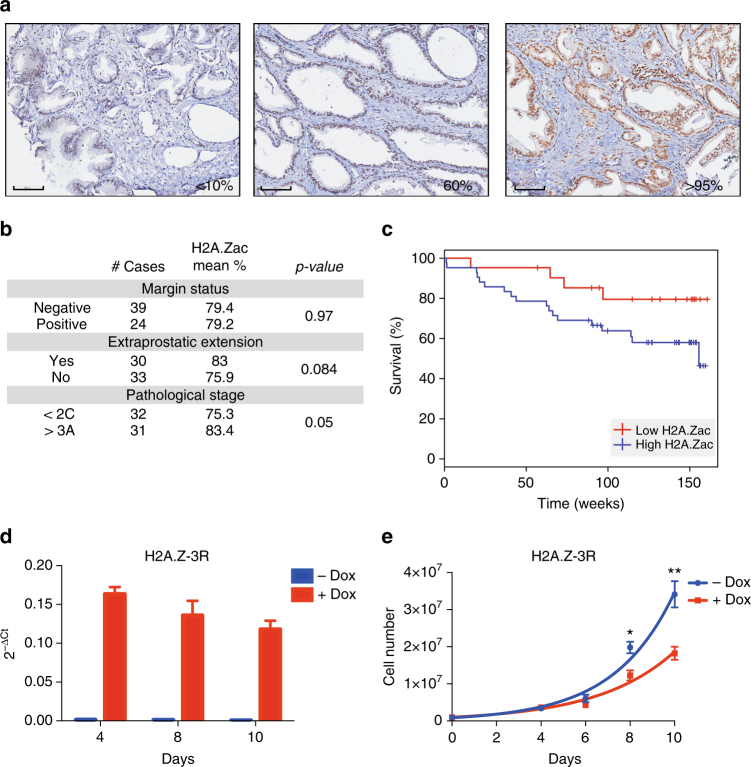
H2A.Zac has pro-oncogenic characteristics in prostate cancer. **a** Immunohistochemical staining of tissue microarrays for H2A.Zac total protein. Tissue microarrays were stained with H2A.Zac specific antibody (brown) and counterstained with hematoxylin (blue). Three representative tumor samples to show the scoring criteria used for differential H2A.Zac protein levels at × 20 magnification (Aperio ImageScope sofware). Left hand site (LHS), low levels, 0–10%; middle panel, moderate (50–65%) and right hand site, (RHS), strong staining (95–100%). Scale bar = 100 μm **b** Summary of the clinical parameters analyzed for H2A.Zac presence in 63 prostate tumor samples. Number of patients and mean value for H2A.Zac presence percentage are shown for each category. The associations between H2A.Zac status and discrete categorical variables were tested using the t-tests. **c** Kaplan-Meier plot of the survival analyses evaluating disease relapse was performed on the nuclear averaged H2A.Zac scores (Wald *p*-value = 0.044). The split into high and low was done on the basis of the median, H2A.Zac high (blue, >=80%) and H2A.Zac low (red, <80%). **d** Real-Time qPCR for the mRNA expression levels of 3R mutant form of H2A.Z in LNCaP cells over 10 days of daily Doxycycline (Dox) treatment. (*N *> 3). Data was normalized using the 2^−ΔCt^ method. Error bars are shown as s.d. **e** Cell proliferation curve of the 3 R cells over 10 days of Dox treatment. Alive cells were automatically counted (*N* > 4) on days 4, 6, 8, and 10 (* *t*-test *p-*value < 0.05 and ** *t*-test *p*-value < 0.001). The graph showed is a representative example of three independent experiments. Error bars are shown as s.d

Global levels of the histone variant H2A.Z are overexpressed in various types of cancer and is associated with poor prognosis^7^. However the levels or prognostic significance of the acetylated form of H2A.Z has not been tested. To determine the global levels of H2A.Zac and relationship with prognosis we performed immunohistochemistry in tissue microarrays from high risk localized prostate cancer cases, using an antibody that recognizes H2A.Z acetyl K4+K7+K11^4^ (Supplementary Table 1, Supplementary Fig. 1a–c, Fig. 1a). We found a significant association between positive H2A.Zac staining tumor nuclei cases (percentage) and poor prognosis with a shorter median biochemical relapse-free survival (Kaplan–Meier plot, Wald *p-*value = 0.04, Fig. 1b and c). There was also correlation between high H2A.Zac and higher tumor stage (t-test *p-*value = 0.05, Fig. 1b).

To further address a pro-oncogenic role of H2A.Zac in prostate cancer, we generated a doxycycline inducible cell model in the prostatic adenocarcinoma cell line LNCaP that expresses a mutant form of H2A.Z-1, whereby the three most commonly acetylated lysines (K4, K7, and K11)^6, 12^ have been replaced by arginines (3R cells, Fig. 1d). This triple mutation (K to R) conserves the positive charge of lysines, but cannot be acetylated^19^, and therefore acts as a dominant negative acetylation H2A.Z mutant. We find that the 3R cells show a gradual decrease in the proliferation rate upon the expression of H2A.Z mutant (+Dox) (Fig. 1d and e) suggesting that H2AZ acetylation may play a role in prostate cancer cell growth.

### Mis-localization of H2A.Zac at active enhancers in cancer

**Fig. 2:**
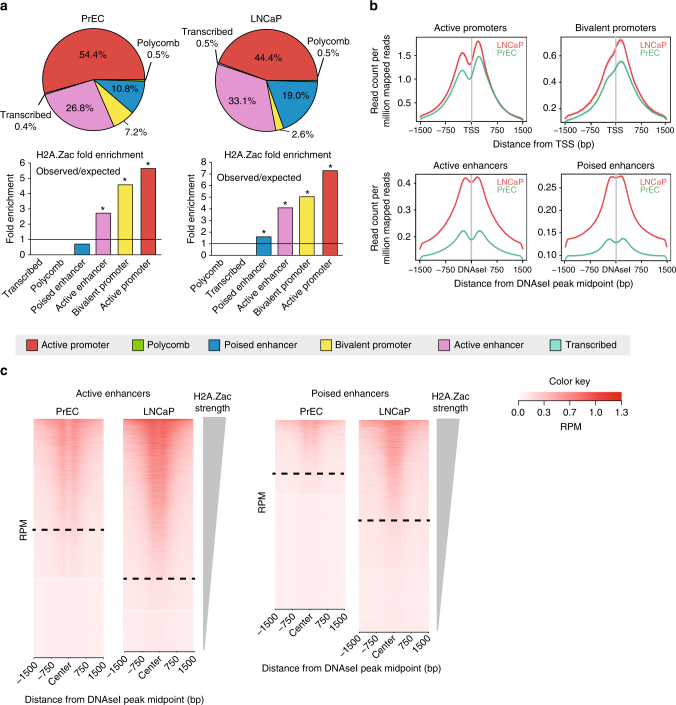
H2A.Zac occupancy at genomic regions in PrEC and LNCaP. **a** Genomic Association Test (GAT) of H2A.Zac ChIP-seq and ChromHMM regions in LNCaP and PrEC cells. A total number of 42,538 H2A.Zac intersecting peaks from biological replicates were detected in LNCaP and 31,479 H2A.Zac peaks in PrEC. Pie charts (upper panel) representing the percentage of marked H2A.Zac peaks falling in each ChromHMM state. Observed vs. expected fold enrichment graphs (lower panel), * *p-*value < 0.0001 of significant enrichment. The line indicates the threshold of the significant enrichment. **b** Ngs plots of the average signal (read count per million mapped reads) of H2A.Zac ChIP seq in LNCaP (red) and PrEC (green) cells for each genomic region of significant enrichment. The plots were centered at the transcriptional start site (TSS) in the case of active and bivalent promoters and at the midpoints of DNase I hypersensitive sites sequencing (DNAseI) peaks in the case of active or poised enhancers. **c** Heatmaps showing H2A.Zac ChIP-seq signal (ordered by H2A.Zac signal intensity from top to bottom) across all the assigned active and poised enhancers, according to ChromHMM. H2A.Zac was centered using DNAseI midpoint. Dashed line separates enhancers as “with H2A.Zac” or “ without H2A.Zac”. Scale bar shows the colorkey of the intensity of H2A.Zac ChIP average signal (read count per million mapped reads, RPM)

We surmised that increased levels of H2A.Zac observed in prostate cancer may reflect a mis-localization of the histone variant modification in chromatin. To address if there is an alteration in the genome-wide distribution of H2A.Zac-associated nucleosomes and therefore a potential regulatory role in cancer, we first profiled the genomic and functional location of H2A.Zac in normal prostate (PrEC) and the androgen hormone sensitive prostate cancer cell line LNCaP by ChIP-seq. To demarcate the different regulatory regions in the normal and cancer cells we used ChromHMM^20, 21^ to define active promoter, bivalent promoter, polycomb, active enhancer, poised enhancer, and transcribed regions^21^ (Fig. 2). We found a significant enrichment of H2A.Zac peaks at active and bivalent promoters and active enhancers in both the normal PrEC cells and LNCaP cancer cells, but notably poised enhancers were enriched only in the cancer cells (Fig. 2a). In addition, the distribution of H2A.Zac at the different regulatory regions was similar between cell lines (Fig. 2b), where H2A.Zac flanks TSS and DNAseI sites at promoters and enhancers, respectively. H2A.Zac ChIP-seq data were also visualized using heatmaps at enhancer regions (Fig. 2c). Interestingly, the H2A.Zac signal was present in a higher number of enhancer regions in LNCaP compared to PrEC at both active and poised enhancers, for instance, ~ 60% of LNCaP active enhancers with H2A.Zac presence vs. ~ 40% of PrEC active enhancers containing H2A.Zac and ~ 50% in LNCaP vs. ~ 20% in PrEC of poised enhancers containing H2A.Zac.

To compare if there was a difference in genome-wide occupancy at regulatory regions between total H2A.Z and H2A.Zac, we also performed total H2A.Z ChIP-seq and downstream ChromHMM analyses in parallel (Supplementary Fig. 2a, b). Total H2A.Z peaks were significantly enriched at promoters and enhancers (Supplementary Fig. 2a). However there was a higher percentage of peaks observed at polycomb marked regions, suggesting a role for non acetylated H2A.Z at repressive chromatin regions, as previously described in ES cells^11, 12^. We found that H2A.Z distribution across the different regulatory regions was similar between the cancer and normal cells (Supplementary Fig. 2b).

**Fig. 3:**
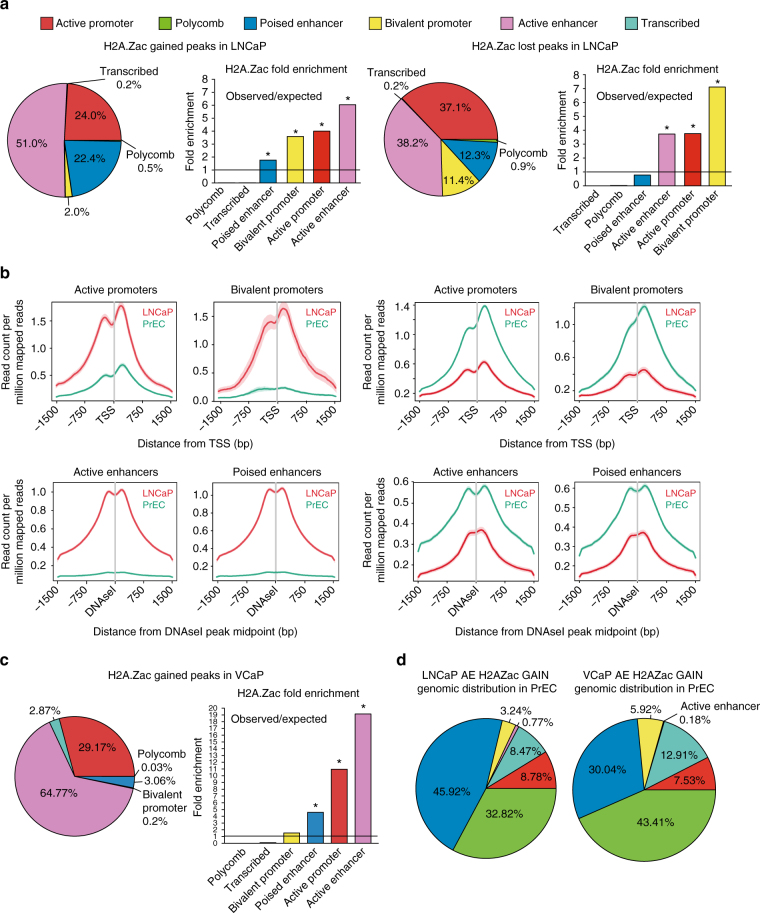
Aberrant H2A.Zac is highly remodeled at enhancers and promoters in cancer. **a** GAT analysis of the gained and lost peaks in LNCaP compared to PrEC at ChromHMM regions. A total number of 3680 peaks (300 bp bins) were gained and 5113 peaks (300 bp bins) were lost in LNCaP compared to PrEC. These peaks were then assigned to the different genomic regions called by ChromHMM; for the gained peaks it was compared with LNCaP ChromHMM and for the lost peaks the PrEC ChromHMM was used. GAT analyses were represented using pie charts (LHS of each panel) and observed vs. expected diagrams (RHS of each panel). * *p-*value < 0.0001 of significant enrichment. The line indicates the threshold of the significant enrichment. **b** Ngs plots of the of the average signal (read count per million mapped reads) of H2A.Zac gained (LHS) and lost (RHS) peaks in LNCaP (red) compared to PrEC (green) for each significant genomic regulatory region. The plots were centered to the transcriptional start site (TSS) in the case of active and bivalent promoters and to the midpoints of DNase I hypersensitive sites sequencing (DNAseI) peaks in the case of active or poised enhancers. **c** GAT analysis of the gained H2A.Zac peaks in VCaP compared to PrEC at VCaP ChromHMM regions. A total number of 6383 peaks (200 bp bins) were gained in VCaP compared to PrEC. These peaks were then assigned to the different genomic regions called by VCaP ChromHMM; GAT analyses were represented using pie charts (LHS) and observed vs. expected diagrams (RHS). * *p-*value < 0.0001 of significant enrichment. The line indicates the threshold of the positive vs. negative enrichment. **d** Pie charts of GAT analyses of LNCaP gained peaks (*n* = 1803, LHS) and VCaP gained peaks (*n* = 3104, RHS) at a neo-enhancers at PrEC ChromHMM regions

To next address the downstream transcriptional and epigenetic consequences associated with changes in H2A.Zac in cancer cells, we identified the significant lost and gained H2A.Zac ChIP peaks in LNCaP cells compared with PrEC cells^22^ and then overlapped the differential binding loci with the different regulatory regions across the genome using LNCaP ChromHMM states for the gained peaks and PrEC ChromHMM states for the lost peaks (Fig. 3a, b). We found the highest percentage of gained H2A.Zac peaks were located at active enhancers (51%) followed by active promoters (24%) and poised enhancers (22.4%) (Fig. 3a, LHS). For lost H2A.Zac peaks, we also found the most change at active promoters (37.1%) and enhancers (38.2%) followed by poised enhancers (12.3%) and bivalent promoters (11.4%). Notably, the lost H2A.Zac peaks at bivalent promoters were the most prevalent in cancer cells (Fig. 3a, RHS). Figure 3b shows the marked differences in H2A.Zac signal between LNCaP and PrEC for the gained and the lost peaks. This data reveal that H2A.Zac nucleosome occupancy is different in cancer cells, notably with increased presence of H2A.Zac nucleosomes at active enhancers, suggesting a key role for H2A.Zac in the generation of new ectopic or prostate cancer neo-enhancers. To further confirm whether the formation of prostate cancer neo-enhancers, upon H2A.Zac gain, could be a general mechanism in prostate cancer, we performed H2A.Zac ChIPseq experiments, as well as ChromHMM in an additional androgen-dependent prostate cancer cell line VCaP. We found that H2A.Zac gained peaks were also most commonly associated with active enhancers (65%, Fig. 3c). Indeed 25% of the neo-enhancers are shared between LNCaP and VCaP cells. (Supplementary Fig. 3b), suggesting that H2A.Zac aberrant gain at active enhancers could be a hallmark of prostate cancer.

**Fig. 4:**
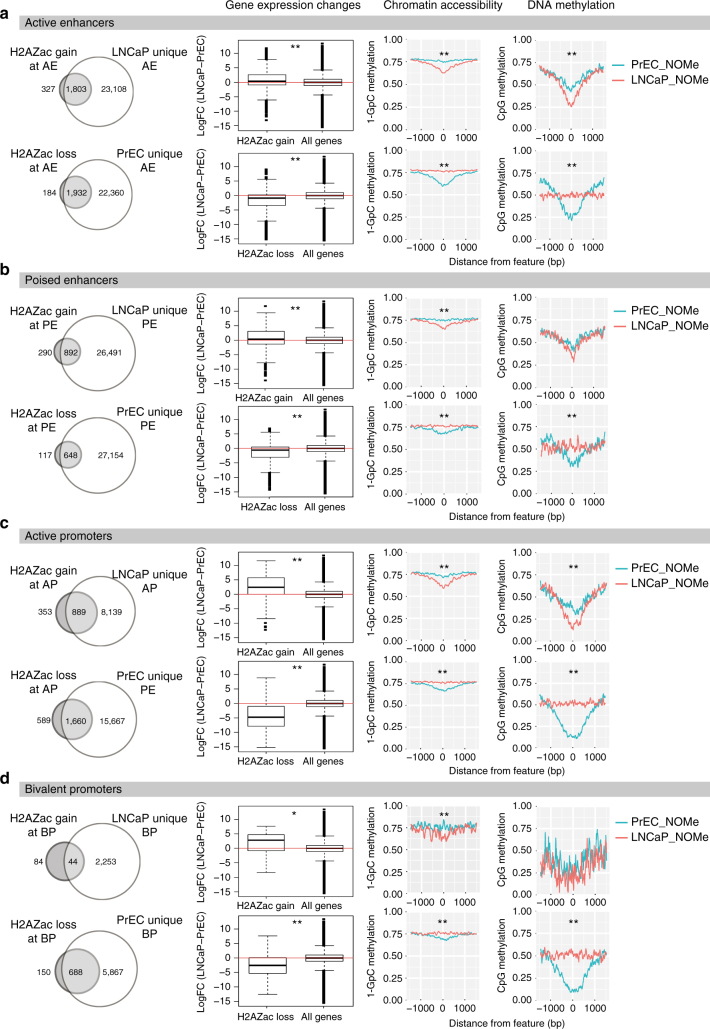
Epigenetic features of H2A.Zac re-distribution at genomic regulatory regions. LHS, Venn diagrams overlapping the H2A.Zac gained or lost peaks from each different regulatory region (from Fig. 3a) with the corresponding “unique” active enhancers **a**, poised enhancers **b**, active promoters **c** and bivalent promoters **d**. The terminology “unique” was used to call enhancers or promoters that were only present in LNCaP but not in PrEC, for the gained peaks; and the enhancers and promoters that were only present in PrEC but not in LNCaP for the lost peaks. Box plots were generated to address the correlation of gain and loss of H2A.Zac peaks at the unique enhancers (active, **a** and poised, **b** or promoters (active, **c** or bivalent **d**) with gene transcriptional regulation. The logarithmic fold change expression (logFC) of LNCaP minus PrEC was compared between all genes and the overlapping regions from the corresponding Venn diagrams. To associate gene expression with enhancers, all the genes upstream or downstream of a particular enhancer within a 25 kb window were assigned as an enhancer-gene association **a** and **b** RHS, two types of NOMe plots were generated to evaluate differential chromatin accessibility, (represented as 1 minus GpC methylation ratio) and differential DNA methylation 0–1 ratio) between LNCaP (red) and PrEC (green). The regions analyzed corresponded to the overlapping areas from the corresponding Venn diagrams for active enhancers **a**, poised enhancers **b**, active promoters **c** and bivalent promoters **d**. * *t*-test *p-*value < 0.05 ** *t*-test *p-*value < 0.001

We therefore asked if the differential H2A.Zac peaks were associated with regulatory regions newly formed or lost in cancer cells. H2A.Zac differential cancer associated peaks were overlapped with the ectopic (unique) epigenetic regulatory regions for each cell line, as determined using ChromHMM (Fig. 4, LHS). Overall, there was a high overlap between cancer specific regulatory regions and a gain or loss of H2A.Zac demonstrating that changes in H2A.Zac nucleosome occupancy correspond to changes in the chromatin state of enhancers and promoters in cancer. Of note, 84% of the gained H2A.Zac peaks are associated with a gain in ectopic active enhancers in LNCaP (Fig. 4a, LHS) and 95% in VCaP (Supplementary Fig. 3a). On the other hand, 91% of the LNCaP and 94% of the VCaP lost peaks overlap with unique active enhancers in PrEC cells (Fig. 4a, LHS and Supplementary Fig. 3a). Notably, H2A.Z associated nucleosomes were not commonly found in PrEC cells (only in 24%) at the neo-enhancers identified in LNCaP cells, suggesting that these loci were formed with incorporation of both H2A.Z and its acetylation state to form the neo-enhancers (Supplementary Fig. 2c).

To further characterise the origin of the prostate cancer neo-enhancers, we determined their functional annotations in PrEC cells using ChromHMM (Fig. 3d). We found that 33% of LNCaP neo-enhancers were marked as polycomb and 46% as poised enhancers in PrEC cells and for VCaP neo-enhancers, 43% were marked as polycomb and 30% poised enhancers. Thus the newly formed neo-enhancers identified in the prostate cancers were either marked by repressive chromatin in normal prostate epithelial cell lines, or corresponded to poised enhancers.

### H2A.Zac associates with epigenetic gene activation

We next asked if the gained and lost H2A.Zac ChIP peaks corresponded to a change in gene expression and to a change in the chromatin characteristics of the cancer epigenome. First, we found that the remodeled H2A.Zac regions correlated with significant changes in gene expression (Fig. 4, box plots). A gain of H2A.Zac at active enhancers (AE), poised enhancers (PE), active promoters (AP), and bivalent promoters (BP) was positively correlated with gene up-regulation in LNCaP cells (*p-*value < 0.0001), while H2A.Zac lost peaks correlated with gene down-regulation (*p-*value < 0.0001) (Fig. 4). Furthermore, the association of gene up-regulation with H2A.Zac redistribution at cancer neo-enhancers was further validated in VCaP cells (Supplementary Fig. 3c). We next performed Gene Set Enrichment Analysis (GSEA) of the genes associated with all the LNCaP and VCaP neo-enhancers and found a remarkably high concordance in the significant gene sets (Supplementary Fig. 3d, highlighted in blue). LNCaP and VCaP neo-enhancers were linked to oncogenic-related pathways including androgen response, a hallmark of prostate cancer^23^, and KRAS signaling, a well-known oncogenic pathway in many cancer types including prostate cancer^24^. Similarly, we found a high concordance in the common pathways in the lost active enhancers between LNCaP and VCaP cells (Supplementary Fig. 3e, highlighted in blue) including genes involved in tumor suppression pathways like TNFalpha signaling^25^. Altogether these results support our finding that H2A.Zac associated activation of prostate cancer neo-enhancers results in a common signature in prostate cancer.

To further characterise the downstream epigenetic effects associated with gain and loss of H2A.Zac, we analyzed DNA methylation changes and chromatin accessibility at these dynamic sites by NOMe-seq^26^. The NOMe plots in Fig. 4 shows that gain of H2A.Zac strongly associates with higher levels of chromatin accessibility at all analyzed regulatory regions (*t*-test *p-*value < 0.001 and conversely loss of H2A.Zac associates with reduced levels of chromatin accessibility (*t*-test *p-*value < 0.01). We also found a strong anti-association of DNA methylation and H2A.Zac occupancy, not only at active promoters^5^, but also at active enhancers (*t*-test *p-*value < 0.001) (Fig. 4, DNA methylation NOMe-plots).

**Fig. 5:**
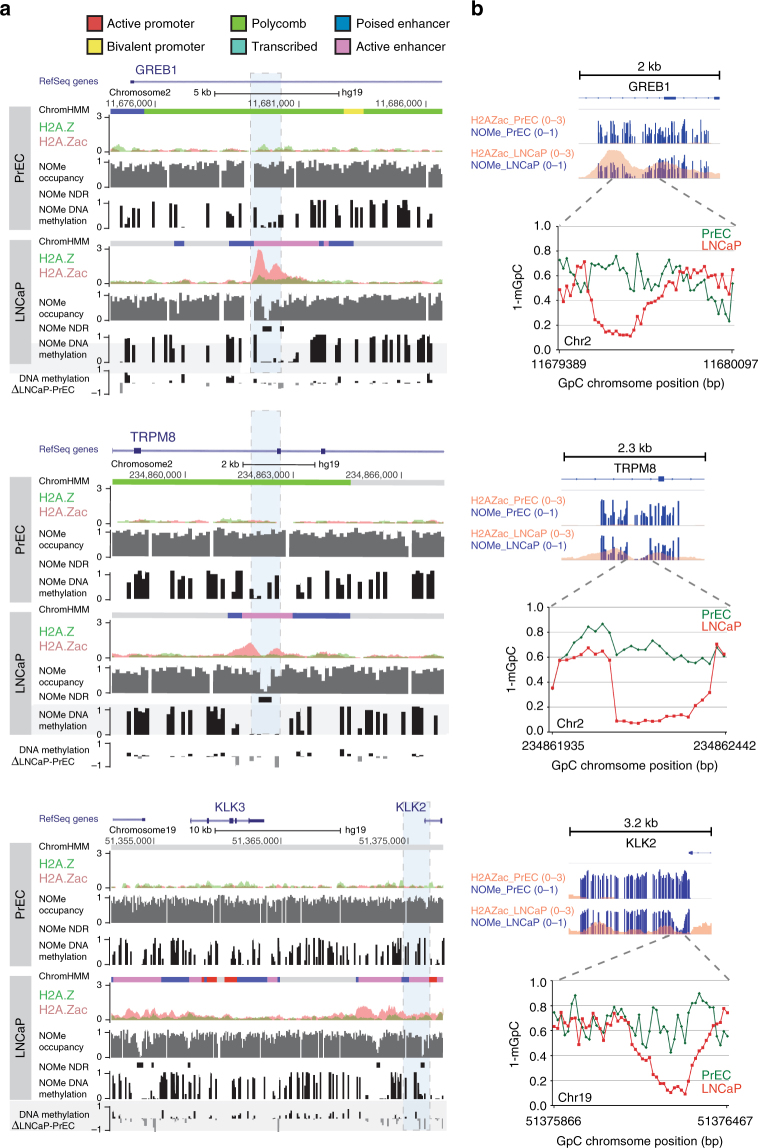
H2A.Zac-promoter and enhancer remodeling of cancer-related genes. **a** University of California Santa Cruz (UCSC) genome browser hg19 screen shots of representative genes that exhibited remodeling of H2A.Zac at promoters and/or enhancers between PrEC and LNCaP. In these screen shots different types of data are presented for both PrEC and LNCaP cell lines (from top to bottom): ChromHMM color-coded according to the different states (see legend); merged tracks of H2A.Z (green) and H2A.Zac (red) ChIPseq signal (RPM); NOMe-seq tracks for chomatin accessibility (NOMe occupancy) in which 1-mGpC ratio (0–1) is represented as a gray bar in 100 bp smoothed resolution. NDRs were called from the NOMe-seq data (with a *p-*value cutoff of −log10(p) > 15) and are represented as black blocks (NOMe NDR); NOMe-seq tracks for DNA methylation (NOMe DNA methylation) in which mCpG ratio (0–1) is represented as a black bar in 100 bp smoothed resolution; Differences in DNA methylation ratio between LNCaP and PrEC (DNA methylation ∆LNCaP-PrEC) were calculated in each 100 bp bin, gray bars represent loss in LNCaP (negative value) and black bars represent gain in LNCaP (positive value). The dashed blue rectangles highlight the regions selected for the validation of the chromatin accessibility by targeted NOMe-seq (section b). All of the selected gene examples are androgen-regulated genes (based on expression array data and AR ChIP-seq) and are also up-regulated in LNCaP compared with PrEC as shown in Fig. S2A. **b** Targeted NOMe-seq validation of chromatin accessibility and its correlation with H2A.Zac presence. Upper panel, merged IGV tracks of PrEC (top) and LNCaP (bottom) cells showing H2A.Zac ChIP-seq signal (orange, 0–3 RPM) merged with targeted NOMe-seq data (dark blue bars blue bars, 0–1, represented as 0–1 ratio of each 1-mGpC site), of two enhancer regions, GREB1 and TRPM8 and one promoter region, KLK2. The bottom diagrams represent a magnification at the identified NDR of each genomic region were the x axis represents each GpC site and the *y* axis is the average ratio of 1-mGpC of each site, in PrEC (green) and LNCaP (red). The regions analyzed by targeted NOMeseq were CpG poor disabling us to obtain a comprehensive map for CpG methylation changes

Notably the concordant epigenetic changes of H2A.Zac nucleosome occupancy, chromatin accessibility, DNA methylation and gene expression, were observed at promoters and enhancers of cancer-related genes that are overexpressed in LNCaP cells (Fig. 5a and Supplementary Fig. 4a) and in clinical prostate cancer, *GREB1, TRPM8, KLK2*, and *KLK3* ^27^. Figure 5a shows examples of the alterations in H2A.Zac occupancy corresponding to changes in chromatin accessibility and DNA methylation. Chromatin accessibility was further validated using targeted NOMe-seq for two enhancer regions (*GREB1* and *TRMP8*) and one promoter region (*KLK2*) (Fig. 5b). This method generates drastically higher resolution mapping (~×10,000) of the regions of interest relative to clonal NOMe-seq^28^ (Supplementary Fig. 4b and c). Altogether the data demonstrate a genome-wide association between H2A.Zac occupancy, accessible chromatin and DNA hypomethylation to facilitate open chromatin conformation and gene activation at cancer-related enhancers and promoters.

### H2A.Zac nucleosomes are remodeled at AR-enhancers

**Fig. 6:**
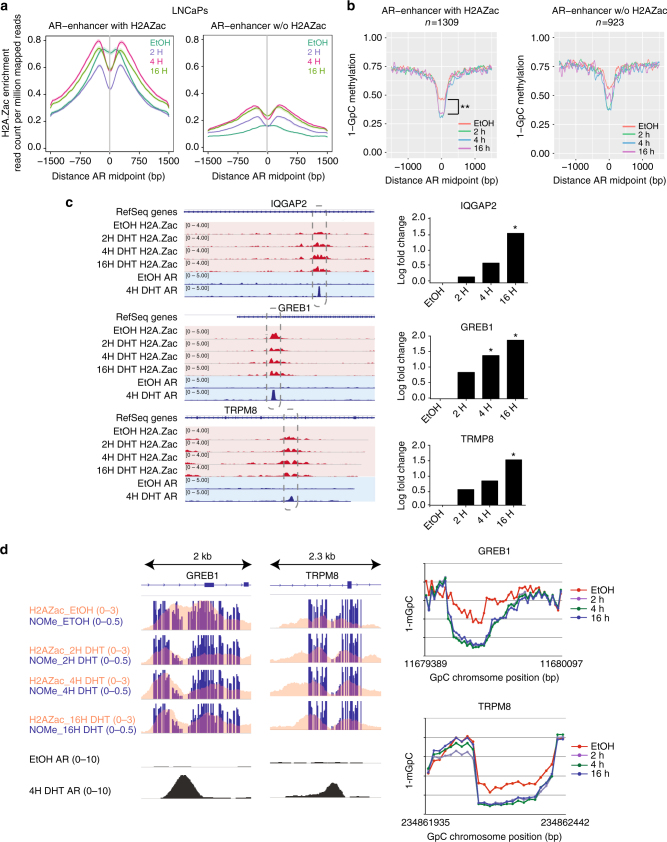
H2A.Zac-nucleosome dynamics occurs at AR enhancers. **a** H2A.Zac ChIP-seq average intensity profile using Ngs plots during a time course of DHT treatment, from the control (EtOH), to the following DHT timepoints: 2 h, 4 h and 16 h in LNCaP cells and control (EtOH). The AR sites were split according to the presence of H2A.Zac at AR enhancers; the peaks that overlapped with LNCaP ChromHMM active enhancers were considered as AR enhancers. **b** NOMe plots representing chromatin accessibility at the AR enhancers with H2A.Zac (LHS) and without H2A.Zac (w/o) (RHS) during the different timepoints of DHT treatment. The dashed lines indicate the maximum accessibility at EtOH (red line) and 16 h (purple line). ** t-test *p-value* < 0.001. **c** LHS, IGV screen shots of the androgen-sensitive genes *IQGAP2, GREB1* and *TRPM8* showing the tracks for H2A.Zac ChIP-seq at EtOH and the different DHT timepoints (red) and AR ChIP-seq (blue) for EtOH and 4 h of DHT treatment. The dashed boxes indicate the AR binding site at the enhancer areas of these genes. RHS, gene expression of these genes across the DHT time course. These data was obtained from Affymetrix Human Gene 2.1 ST expression arrays and it is represented as logarithmic fold chance. *adjusted *p-*value < 0.05. **d** LHS, merged IGV tracks of control (EtOH) and DHT timepoints (2 h, 4 h and 16 h) showing H2A.Zac ChIP-seq signal (orange, RPM) overlapped with targeted NOMe-seq average signal, represented as 0–1 ratio of each 1-mGpC site (dark blue bars), of two AR enhancer regions for *GREB1* and *TRPM8* genes. The bottom two tracks correspond to AR ChIP-seq (gray, RPM) to show how the increase of accessibility correlates with AR binding. The diagrams at the RHS represent a magnification at the identified NDR of each genomic region where the x axis represents each GpC site and the y axis is the average ratio of 1-mGpC of each GpC site, in the DHT timecourse

To determine the temporal epigenetic changes and H2A.Zac remodeling at AR associated gene promoters and enhancers during gene activation, H2A.Zac ChIP-seq, gene expression arrays, and NOMe-seq were performed in LNCaP cells treated with 5alpha-dihydrotestosterone (DHT) for different timepoints (2 h, 4 h, and 16 h). In addition, AR sites were determined using a public AR ChIP-seq data set (Supplementary Table 2). To investigate the chromatin dynamics at AR sites we split the AR sites according to their association with (w-H2A.Zac) or without H2A.Zac (wo-H2A.Zac) at non-induced conditions (EtOH). We found that ~ 50% of AR peaks contain H2A.Zac (Supplementary Fig. 5a). As previously described^16, 17^, AR sites are mostly found at active enhancers (Supplementary Fig. 5b), and of these ~ 60% co-occur with H2AZac (Fig. 6a, LHS) and 40% are not associated with H2AZac (Fig. 6a, RHS). Under non-induced conditions H2A.Zac nucleosomes span at the AR enhancer sites; however, upon androgen stimulation H2A.Zac nucleosomes are rapidly redistributed to flank the AR binding sites. Similar redistribution of H2A.Zac nucleosomes to flank the AR-enhancer binding sites after 16 h DHT treatment was observed in VCaP cells (Supplementary Fig. 5c).

Next we used the NOMe-seq data to simultaneously study temporal changes in chromatin accessibility and DNA methylation at AR enhancers and its association with H2A.Zac. AR sites show a grade of accessibility before induction (Supplementary Fig. 5d, LHS), as previously reported^29^, consistent with poised enhancers. DNA was also hypomethylated in the absence of androgens and remained unmethylated during androgen induction (Supplementary Fig. 5d, RHS). When we compared AR w-H2A.Zac sites with AR w/o-H2A.Zac we found the enhancers containing H2A.Zac become significantly more accessible at 16 h of DHT induction (*p-*value = 0.0009) than enhancers w/o-H2A.Zac (*p-*value = 0.13), (Fig. 6b). As early as 2 h after treatment, the chromatin then becomes “fully” accessible, with no further changes in accessibility at later timepoints and always keeping the level of accessibility higher at the AR w-H2A.Zac enhancers, even under non-induced conditions (Fig. 6b and Supplementary Fig. 6a). In addition, we did not observed notable differences in DNA methylation temporal changes between AR w- and w/o-H2A.Zac, where both AR groups where highly hypomethylated (Supplementary Fig. 5e).

Figure 6c highlights specific examples of the dynamics of H2A.Zac remodeling at AR-enhancers of known androgen-induced genes, *IQGAP2*, *GREB1*, and *TRPM8* and Fig. 6d shows two specific examples at *GREB1* and *TRPM8* AR-enhancers validated by targeted NOMe-seq.

The data suggest that H2A.Zac nucleosomes are either more unstable or are associated with a more active nucleosome turnover in order to “guide” AR and facilitate nucleosome eviction or repositioning in AR sites.

Gene expression arrays were also performed at each timepoint. There were no significant expression changes observed at 2 h of DHT induction (FDR < 0.05) and only 51 genes were significantly changed at 4 h. The majority of the expected changes in gene expression were observed at 16 h (777 known genes), demonstrating that the observed chromatin changes in nucleosome positioning and H2A.Zac remodeling at AR enhancers occur prior to gene transcription. As H2A.Zac is also a key chromatin marker for active promoters, we therefore next asked if H2A.Zac remodeling occurs during the transition from poised to active promoter. We plotted the H2A.Zac active promoter signal for genes that were up or down-regulated at 16 h of DHT induction and found no significant changes in H2A.Z signal or profile at the promoters of any of androgen-regulated genes (Supplementary Fig. 6b). In fact androgen-regulated genes showed already high enrichment of H2A.Zac flanking the TSSs at their promoters in the poised state (EtOH). Concordantly, there was no change in chromatin accessibility and no difference at the TSS of the androgen-regulated genes, as all the genes were already marked by NDRs under non-induced conditions, suggesting a pre-existing poised or bivalent state (Supplementary Fig. 6c). Targeted NOMe-seq at KLK2 gene promoter showed subtle differences in chromatin accessibility that were not observed genome-wide (Supplementary Fig. 6d). Altogether, we found that H2A.Zac nucleosomes are associated with more accessible poised or active AR-enhancers and that H2A.Zac is remodeled at AR-enhancers upon androgen activation before gene transcription changes, suggesting that H2A.Zac is a pre-requirement for the formation of poised chromatin.

### H2A.Zac-AR enhancers have hallmarks of active enhancers

**Fig. 7:**
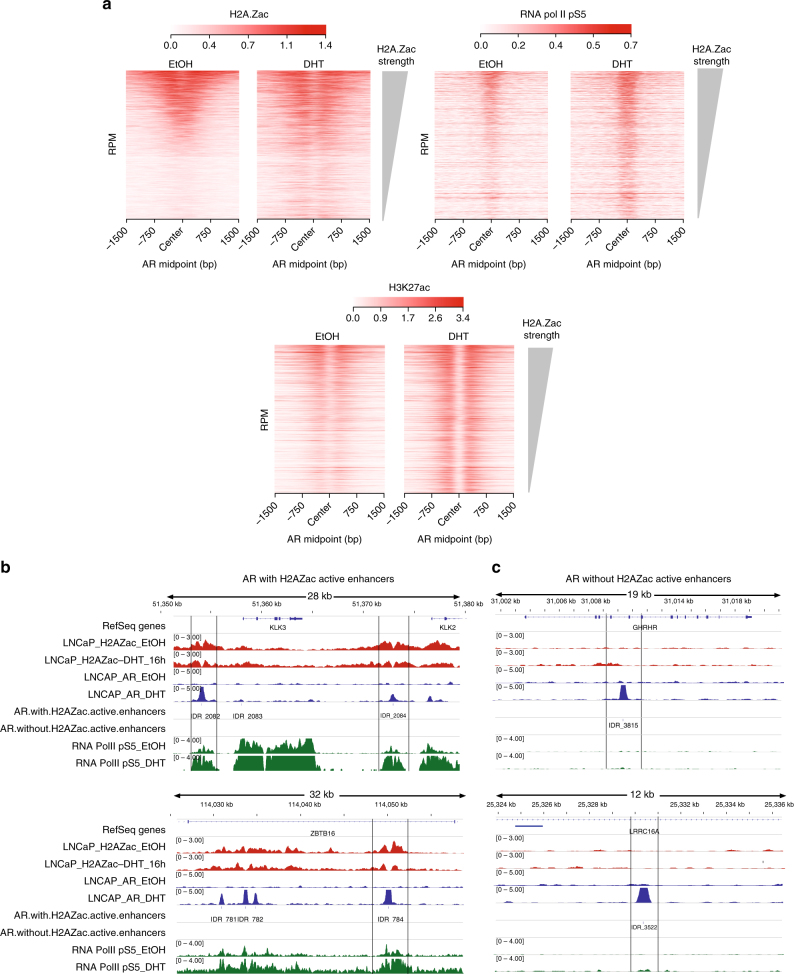
AR enhancers flanked by H2A.Zac-nucleosomes have features of active enhancers. **a** Heatmap of H2A.Zac (LHS), RNA polymerase II phospho S5 (RNA pol II pS5, center) and H3K27ac (RHS) ChIP-seq data at AR enhancers (*n* = 2232) ordered by H2A.Zac signal intensity from highest (top) to lowest at starving conditions (EtOH) and DHT treatment (DHT). Scale bars show the colorkey of the intensity of each corresponding ChIP average signal (RPM). **b** IGV screen shots of representative examples for AR active enhancers with H2A.Zac (LHS) or **c** without H2A.Zac (RHS) (highlighted in a rectangle) showing H2A.Zac (red), AR (blue) and RNA pol II pS5 (green) ChIP-seq signal at these sites (RPM)

**Fig. 8:**
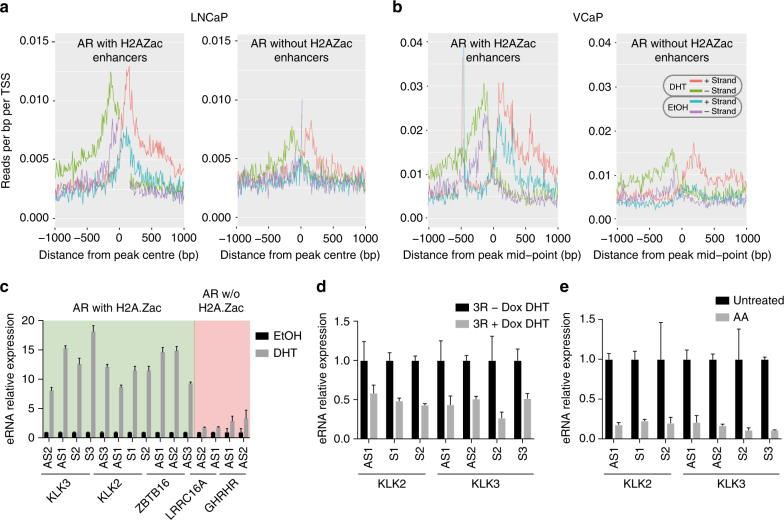
H2A.Zac is involved in androgen-dependent eRNA expression. Bidirectional histograms (+and – strands) of eRNA profile from GROseq data around AR enhancers during starvation (EtOH) and after DHT treatment (DHT) in the androgen responsive prostate cancer cell lines LNCaP **a** and VCaP **b**. AR ChromHMM-defined active enhancers were split into with/without H2AZac and centered on AR peaks as per Fig. 6a, obtaining 1309 and 808 overlaps for AR-enhancers with H2A.Zac and 923 and 1264 overlaps for AR-enhancers without H2A.Zac in LNCaP and VCaP, respectively. Note, four replicates were done for LNCaP GROseq and two replicates for VCaP GROseq, these plots represents one representative replicate. **c** Real Time qPCR for androgen-responsive eRNA expression in LNCaP cells during starvation (EtOH) or 16 h of DHT treatment (DHT) of representative examples from AR active enhancers flanked by H2A.Zac-nucleosomes (AR with H2A.Zac, green background) or not flanked (AR w/o H2A.Zac, red background) (see Fig. 7b). Data is presented as the fold change in eRNA expression normalized to the expression level in EtOH condition (*N* = 3). **d** Real Time qPCR for androgen-responsive eRNA expression of representative eRNAs at AR enhancers flanked by H2A.Zac (*KLK2* and *KLK3* eRNAs, (Fig. 7b) in 3R cells during 16 h of DHT treatment (DHT) and daily Doxycycline (Dox) stimulation (+) or non stimulation (−). Data is shown as the fold change in eRNA expression normalized to the expression level in –Dox DHT condition (*N* = 2). **e** LNCaP cells were either untreated or treated (AA) with Anacardic Acid at 90 µM for 48 h (*N* = 2). eRNA expression levels of the AR-enhancer for *KLK2* and *KLK3* genes were measured by Real Time qPCR. Data are shown as the fold change in eRNA expression normalized to the expression level in the Untreated condition. For the eRNA Real time qPCR several primers were used per eRNA at regions around 200–1000 bp from the center of the AR binding site on the sense (S) or anti-sense strand (AS) (See Supplementary Table 4 for primer description). All error bars are shown as s.d

AR requires interaction with its co-factors to activate the androgen-transcriptional program^30^. We therefore asked if H2A.Zac nucleosomes that flank AR-enhancers are associated with any of the AR co-factors with a known oncogenic role in prostate cancer, including CtBP1^31^, CtBP2^32^, FOXA-1, and GATA-2^33^. We used public ChIP-seq data from these AR co-factors performed on LNCaP cells untreated (EtOH) and treated with DHT (Supplementary Table 2), to compare with our H2A.Zac ChIP-seq data. CTBP1, CTBP2, FOXA-1, GATA-2 ChIP-seq signals were ordered by H2A.Zac intensity strength to determine if there was any pattern of association between AR cofactors and H2A.Zac presence at AR enhancers, however, no differences were observed (Supplementary Fig. 7a). We then asked if there was a difference associated with enhancer activity. Active enhancers recruit RNA Pol II^34^, encode for eRNAs^18^ and are marked by both H3K27ac and H3K4Me1^14^. We therefore used the same approach to look for an association between RNA Pol II, eRNA, H3K27ac, H3K4Me1 (Supplementary Tables 2 and 3) and H2A.Zac. Interestingly, we found a higher signal for RNA Pol II phospho S5 (pS5) and H3K27ac enrichment at either starvation or DHT induction conditions at AR enhancers with high H2A.Zac signal (Fig. 7a). A similar trend was also observed in VCaP cells (Supplementary Fig. 7b). In contrast, the H3K4me1 mark shows an even enrichment across all AR enhancer regardless of H2A.Zac presence or absence (Supplementary Fig. 7a). Figure 7b, c shows particular examples of AR enhancers with H2A.Zac (w-H2A.Zac, Fig. 7b) and without H2A.Zac (w/o-H2A.Zac, Fig. 7c) demonstrating noticeable differences in RNA pol pS5 at EtOH and 16 h DHT. Next, we analyzed androgen-dependent eRNA production using GRO-seq public data in LNCaP and VCaP androgen-dependent prostate cancer cell lines (Supplementary Table 3). We observed that there was a higher increase in eRNA transcription upon DHT induction at the AR w-H2A.Zac enhancers compared to the AR w/o-H2A.Zac in both cell lines (Fig. 8a, b). To confirm these differences we also performed subsampling of the AR w-H2A.Zac to have a comparable number of regions and we found the similar higher enrichment with H2A.Zac nucleosomes compared to the eRNAs at AR w/o-H2A.Zac regions (Supplementary Fig. 7c and d). RT qPCR of representative eRNAs from each group (Fig. 8c) was also performed and the same trend was observed, where the AR enhancers flanked by H2A.Zac nucleosomes displayed higher eRNA expression upon DHT treatment.

To determine the functional implications of loss of H2A.Z acetylation, we first tested if the expression of eRNAs was altered in the 3R mutant cells, where H2A.Z acetylation is genetically modified (Fig. 8d). We found a reduction in *KLK3* and *KLK2* eRNA expression, which were previously found to be flanked by H2A.Zac nucleosomes in LNCaP cells (Fig. 7b). Second, we treated LNCaP cells with Anacardic acid (AA), which is histone acetyl transferase (HAT) inhibitor, that has previously be found to reduce acetylation of H2A.Z^5^. Here, we observed a decrease in expression of *KLK2* and *KLK3* eRNAs upon AA treatment (Fig. 8e), and corresponding reduction of H2A.Zac levels was also observed at these AR enhancers (Supplementary Fig. 7e). Altogether, the data suggest that AR-H2A.Zac associated enhancers are more functionally active and that H2A.Zac is directly involved in the facilitation of AR-dependent eRNA transcription at enhancers.

## Discussion

Prostate cancer is the second most common type of cancer and the fifth leading cause of cancer-related death in men^35^. However a complete understanding of the molecular causes of prostate cancer remains elusive. Here we report that a higher percentage of positive staining nuclei for acetylated histone variant H2A.Z is associated with poor prognosis in prostate tumors. While histone variants and their chaperones have emerged as critical players in cancer biology^7^, the mechanistic understanding of their role in carcinogenesis remains limited. Our study supports a causative pro-oncogenic role for H2A.Zac in promoting gene deregulation through redistribution of acetylated H2A.Z nucleosomes in the cancer genome to create neo-enhancers resulting in aberrant gene activation.

Even though overexpression of total H2A.Z in different types of cancer and its role in gene transcriptional deregulation has previously been reported^2, 7^, the specific genomic location and cancer associated function of acetylated H2A.Z has not been addressed. We previously described that H2A.Zac is present at promoters of highly expressed genes and that deregulation of gene expression in prostate cancer is associated with H2A.Zac remodeling at gene promoters^5^. Our new genome-wide data reveals that H2A.Zac is also remodeled at distal regulatory regions (poised and active) and that the change in H2A.Zac nucleosome distribution at enhancers is more pronounced than the changes at gene promoters (Fig. 3). The observed elevated nuclear H2A.Zac staining in prostate cancer, related to poor prognosis, may therefore be due to aberrant chromatin re-localization of the acetylated histone variant. We found that re-localization occurred predominately at newly formed or neo-enhancers, which in turn promotes higher chromatin accessibility, DNA hypomethylation and deregulation of gene expression. Notably, we also found that in response to androgens, in two independent prostate cancer models, there was a dynamic re-organization of H2A.Zac at AR enhancers to promote RNA pol II recruitment and transcription of eRNAs before gene activation, demonstrating that H2A.Zac plays a critical role in priming the functional activity of enhancers essential for oncogene expression. Our data supports previous gene-candidate approaches that have shown expression of the oncogene Cyclin D1 requires H2A.Z acetylation at enhancer and promoter elements in ER positive MCF-7^36^ and ER-negative MDA-MB231^37^ breast cancer cell lines.

**Fig. 9:**
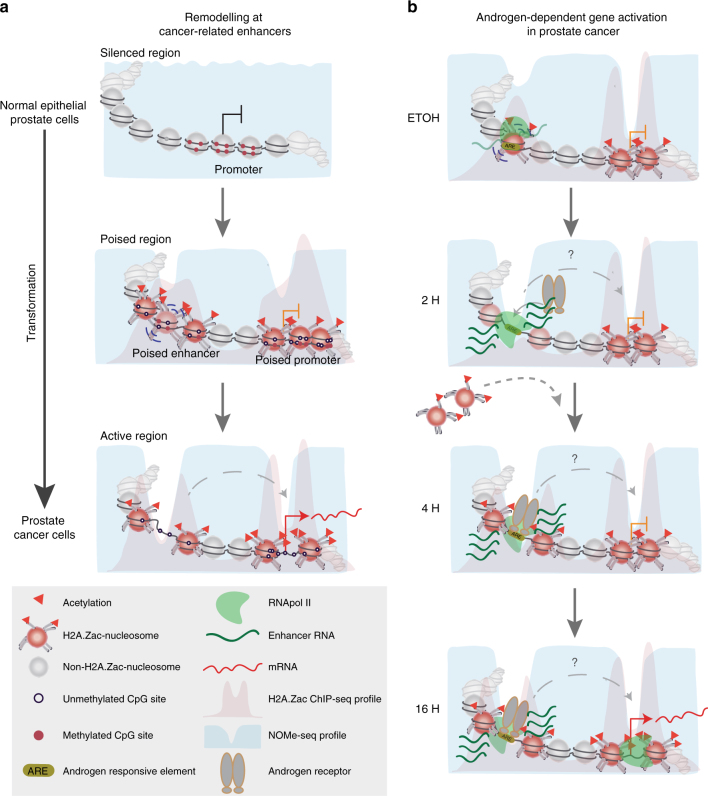
Proposed model for H2A.Zac-enhancer regulation in cancer and androgen signaling. **a** Proposed model of regulation of cancer –activated genes. In a normal context, these genes are silenced and their promoters and enhancers do not have H2A.Zac-containing nucleosomes (Silenced region). During transformation, these genes gain poised epigenetic marks that include a gain of H2A.Zac, loss of DNA methylation and gain of chromatin accessibility at both promoters and enhancers (Poised region). In prostate cancer these genes become activated by an increase of H2A.Zac and a change in its localization from the TSS and +1, +2 nucleosome to flank the TSS at promoters. H2A.Zac is also increased at enhancers and it changes from occupying to flank NDRs. These changes are also associated with a decrease in DNA methylation. **b** Proposed model for H2A.Zac remodeling during androgen signaling. Androgen-sensitive genes remain in a poised state during androgen deprivation (EtOH). This state is characterized by the presence of H2A.Zac nucleosomes at 60% of all the AR enhancer sites and H2A.Zac presence at the nucleosomes flanking the TSSs of all the androgen-sensitive genes. This subset of AR enhancer sites is also characterized by being more accessible. Upon AR activation through DHT treatment (2H, 4H, and 16H), H2A.Zac is removed/shifted to flank AR sites and allow AR binding. This is a very rapid process and occurs prior to gene activation and it is associated with a gain in chromatin accessibility at the AR enhancer sites. We also speculate that this remodeling facilitates the looping formation between gene promoters and AR-enhancers for gene activation

Altogether our findings have led us to propose a new model for aberrant gene transcriptional activation in prostate cancer (Fig. 9a). H2A.Zac-nucleosomes are absent in closed/inactive chromatin at both distal enhancers and gene promoters to ensure appropriate oncogene silencing in normal cells. During transformation H2A.Zac-nucleosomes are recruited or involved in the formation of new regulatory elements, thereby promoting a poised local chromatin conformation. H2A.Zac occupied nucleosomes favor the formation of NDRs and a loss of DNA methylation at both enhancers and promoters, priming these new sites for gene transcription upon androgen stimulation, potentially through enhancer-promoter chromatin looping.

AR is important for prostate cancer development and progression through the regulation of AR target genes involved in prostate cancer growth^23, 38^. The majority of AR binding sites are located at distal regions, and at least some of them are important for transcriptional regulation, and have been identified as “AR-enhancers”, characterized by the presence of HATs, RNA Pol II, eRNAs, H3K4Me1, H3K27ac, NDRs, and H2A.Z^16, 18, 39^. Here we demonstrate that H2A.Zac is remodeled at functionally active AR-enhancers. Figure 9b shows a model to explain our data whereby androgen treatment results in androgen-dependent gene activation at H2A.Zac-AR enhancers and promoters. Notably, H2A.Zac-AR distal regions are characterized by higher chromatin accessibility and a “stalled” RNA Pol II under non-induced conditions. Upon DHT activation, there is specific remodeling of H2A.Zac at AR-enhancers from a poised to an active state and expression of eRNA. Notably expression of eRNAs is reduced in cells that are compromised for acetylated H2A.Z either by genetic manipulation or by treatment with a HAT inhibitor. A recent publication^15^ has also shown that H2A.Z is an important regulator of estrogen receptor enhancer activity, and is required for RNA Pol II and RAD21 recruitment and eRNA transcription suggesting that H2A.Z is also acetylated at estrogen receptor enhancers.

AR-distal regulatory action potentially occurs through chromatin looping^40^. In fact eRNAs may play a role in the initiation or stabilization of enhancer-promoter looping in AR signaling, as demonstrated in particular gene examples^39^. A role for H2A.Z^15^ and H2A.Zac^36, 37^ in chromatin looping has previously been suggested primarily based on gene-candidate studies. Thus we hypothesize that the features of AR-H2A.Zac enhancers suggest that H2A.Zac is involved in enhancer-promoter chromatin looping in order to regulate gene transcription. Future functional studies reporting the causative role of the acetylation of H2A.Z at enhancers are required to further understand how enhancers are regulated during androgen signaling in prostate cancer.

In addition, it is interesting to speculate that the increased chromatin accessibility afforded by H2A.Zac occupancy at distal regulatory regions may be a contributing factor for disease-causative mutations located at enhancers identified in recent GWAS studies^41^. In this context, GWAS studies have identified functional enhancers at genomic regions for high risk prostate cancer^42^. Super enhancers have also been shown to be “hot spots” of disease-associated variation, in particular, oncogenes and other cancer-related genes showed new super-enhancers generated in cancer cells^43^. However more functional studies and correlation analysis in clinical samples are needed to validate the potential association of H2A.Zac with super-enhancers and increased mutational burden.

It has recently been suggested that the isoform H2A.Z.2 likely acts together with histone acetylation to recruit co-activators and transcription factors such as BRD2 and E2F1^44^. Moreover recruitment of BRD2 to the AR regulated genes in prostate cancer has been found to be dependent on H2A.Z.1^45^. Even though these studies did not investigate the acetylation state of the histone variant isoforms it is likely that H2A.Z variants were also modified and is important for BRD2 recruitment. Our study therefore may hold therapeutic potential, as the inhibition of Brd proteins (BET inhibitors) were shown to be effective in inducing cell death in castration-resistant prostate cancer^46^. In summary our study demonstrates that H2A.Zac nucleosome incorporation plays a key role in facilitation of ectopic activation of AR-enhancers, which has major implications regarding the mechanism of oncogene deregulation and potential chromatin therapy options for prostate cancer.

## Methods

### Cell lines and treatments

LNCaP prostate cancer cells (ATCC # CRL-1740) and PrEC normal prostate epithelial cells (Cambrex Bio Science Cat. No. CC-2555: PrEC_1_ tissue acquisition no. #13683) were cultured, as described previously^5^. VCaP (ATCC # CRL-2876) prostate cancer cells were cultured, as described previously^47^. All cell lines were authenticated by STR profiling (CellBank Australia, Westmead, NSW, Australia) and cultured for <6 months after authentication. Mycoplasma contamination testing was routinely performed in the cell lines while in culture (MycoAlert™ Mycoplasma Detection Kit, Lonza).

LNCaP or VCaP cells were seeded at 60–70% confluence and starved with phenol red-free RPMI Medium 1640 containing 5% charcoal-dextran stripped Fetal Bovine Serum (FBS) (LNCaP) or DMEM containing 2.5% charcoal-stripped FBS (VCaP) for 72 h prior to a 2 h, 4 h, or 16 h (LNCaP) or just 16 h (VCaP) treatment with 10 nM 5-α-dihydrotestosterone (DHT; Sigma Aldrich #A8380) or ethanol-treated control. LNCaP cells were treated with Anacardic Acid (6-pentadecylsalicylic acid, AA; Calbiochem) at 90 µM for 48 h. The untreated controls were mock treated with the equivalent amount of the vehicle used to dissolve the drugs, 100% DMSO.

### H2A.Z-3R mutant generation and proliferation assays

The plasmid construct of H2A.Z-1 bearing three mutations at the N terminal (R4, R7, and R11, Supplementary Fig. 1e) was purchased to GeneArt® (Thermo Fisher) and sublconed into the doxycycline inducible system pHUSH-ProEX vector^48^ used as a retrovirus. The construct design was done using degenerated nucleotides in order to distinguish the mutant vs. the endogenous H2A.Z by qPCR (See primers in Supplementary Table 4). Murine ecotropic retrovirus containing H2A.Z-3R PHUSH vector were transiently transfected (FuGENE® 6 Transfection Reagent, Roche) and harvested from ecotropic packing cell line, Platinum E Retroviral Packaging Cell Line^49^ and transduced into LNCaP cells stably expressing the murine ecotropic receptor and select the positive cell clones through puromycin selection (1 µg mL^−1^), as previously described^50^. Positive LNCaP cells were expanded and subjected to daily doxycycline treatment (0.1 µg mL^−1^) to induce H2A.Z-3R overexpression. RT-qPCR (Fig. 1d, Supplementary Table 4) and mass spectrometry were performed to validate the 3 R cell model at mRNA and protein level, respectively.

For cell proliferation assays, 1.0 × 10^6^ of H2A.Z-3R cells were seeded in T75 flasks in RPMI media 10% FBS with (−Dox) or without Doxycycline (+Dox). Cells were counted every other day (4, 6, 8, and 10 days) using the Countess Automated Cell Counter (Invitrogen). Cells were split to always keep ~ 70% confluence. T-test analyses between +Dox and −Dox for each day were performed.

### Patient samples

A cohort of 64 patients with prostate cancer was used in this study. All patients were treated with radical prostatectomy for localized prostate cancer at St. Vincent’s Hospital, Sydney. All surgeries were performed by 1 of 6 specialist urologists. Patients who received neoadjuvant hormonal therapy were excluded from the study. Patients were followed postoperatively by their surgeons on a monthly basis until satisfactory urinary continence was obtained and then at 3-month intervals until the end of the first year, at 6-month intervals to 5 years and yearly thereafter. Relapse was defined by the following criteria: biochemical disease progression with a serum PSA concentration at or above 0.2 ng mL^−1^ rising over a 3-month period or local recurrence on digital rectal examination confirmed by biopsy or by subsequent rise in PSA. This project was approved by the St. Vincent’s Campus Research Ethics Committee (12/231). Informed consent was obtained from all human participants.

### Tissue microarray construction and immunohistochemistry

Tissue microarray construction has been described previously^51^. Briefly, pathologic evaluation of all blocks from each prostate was performed by 1 of 2 histopathologists. Tumors were classified according to the tumor, nodes, metastases (TNM) staging and Gleason grading systems and low-density arrays containing ~ 56 elements (2 mm diameter cores) were created using Kononen et al. technique^52^. Each patient case was represented by a mean of 3 biopsies (range, 2–5 biopsies) of prostate cancer of different primary Gleason score and 1 biopsy of hyperplasia adjacent to cancer. In total, 277 elements representing 64 patients were placed in 7 tissue microarrays.

Immunohistochemistry of acetylated histone H2AZ was performed in these seven tissue microarrays. H2A.Zac antibody validation for IHC was performed doing serial dilutions of the primary antibody and two time-points for antigen retrieval conditions in prostate cancer tissues (Supplementary Fig. 1a–c) As a positive tissue control, additional samples of breast cancer^53^ were included and normal sheep IgG antibody (Santa Cruz Biotechnology sc-2717) was used in parallel as a technical negative control (Supplementary Fig. 1b, c). We also performed in parallel dot blot analysis using H2A.Z recombinant peptides to validate the specificity of H2A.Zac antibody towards the acetylated form (Supplementary Fig. 1d).

Sections were dewaxed in xylene, rehydrated in ethanol grading. Antigen retrieval was performed using the heat-induced epitope retrieval method in a pressure cooker for 60 s in citrate buffer, pH 6.0 (S1699. Dako, Carpinteria, CA), peroxidase quenching was performed with 3% H2O2. The primary antibody was incubated for 1 h, 1:200 H2A.Zac antibody (Abcam #ab18262) or 1:160 normal sheep IgG antibody (Santa Cruz Biotechnology sc-2717), followed by 15-min incubation with 1:200 of the secondary HRP-Polyclonal Rabbit Anti-Sheep antibody (Cat# P 0163, Dako, Carpinteria, CA). A detection system using 3,3′-diaminobenzidine as substrate (Dako) was subsequently used, followed by counterstaining using hematoxylin (Dako).

### Immunohistochemistry assessment and statistical analysis

Immunostaining was independently assessed by three histopathologists (J.G.K.; K.M.L.L. & M.Q.). All individuals were blinded to patient outcome. Nuclear staining was observed, consistent with the known biology function of H2A.Z. Nuclear immunoreactivity was scored as a percentage of the total number of epithelial cells. Percentage scores from the three pathologists were combined into overall percentage scores by using the most common score or (if all three were different) the median score. Slides/Cores with percentage scores that differed by > 30% between pathologists were rescored by two pathologists in a consensus fashion.

For the analysis of H2A.Zac prognostic value, cores without cancer tissue present were removed from data set, leaving a total of 196 slides for 63 individual patients. Each patient had between 1 and 6 slides. Average scores for percentage were computed per patient and used in all further analyses. Biochemical relapse was identified as the event of interest, and the time of the event was taken to be the minimum of the period disease free and time since radical prostatectomy. Given the low sample numbers, categories of other clinical variables were combined. Specifically margin (negative vs others); extracapsular (no vs rest), pathological stage (2 vs 3/4). Gleason score was made into 3 categories < = 6, =7, and >7. A summary of the 63 patient clinical characteristics is presented in Supplementary Table 1.

Survival analyses evaluating disease relapse were performed on the nuclear averaged H2A.Zac scores. Cox proportional hazards models were built, both univariable and multivariable, to assess the significance of the percentage of H2AZac staining taking into account time of relapse. For this model the “y” variable was both the time that had passed since surgery and whether or not a relapse had occurred; and the “*x*” variable, on the other hand, was the percentage of H2A.Zac staining used as a continuous variable. The Wald *p-*value for the univariable case was 0.044; the likelihood ratio test had a *p-*value of 0.021 and log-rank *p-*value was 0.0598. Variables with a *p-*value < 0.05 (in univariable Cox Models) were considered statistically significant and were examined by Kaplan–Meier curves.

Multivariable models assessed H2A.Zac status with other baseline covariates of clinical relevance such as Gleason score, pathologic stage, adjuvant therapy, and preoperative PSA, which were modeled as dichotomous or continuous variables as appropriate. The associations between H2A.Zac status and discrete categorical variables (extraprostatic extension, margins and stage) were tested using *t*-tests. A *p-*value of <0.05 was required for significance and *p-*value < 0.1 was considered as trend. All reported *p*-values are two-sided. All statistical analyses were performed using the R software package.

### Chromatin immunoprecipitation and sequencing

ChIP assays were carried out according to the manufacturer’s protocol (Upstate Biotechnology). Briefly, ~ 2 × 10^6^ cells were fixed by adding formaldehyde at a final concentration of 1% and incubating for 10 minutes at 37 °C. The cells were washed twice with ice cold PBS containing protease inhibitors (1 mM phenylmethylsulfonyl fluoride (PMSF), 1 μg mL^−1^ aprotinin and 1 μg mL^−1^ pepstatin A), harvested and treated with SDS lysis buffer for 10 min on ice. The resulting lysates were sonicated to shear the DNA to fragment lengths of 200–500 bp. The complexes were immunoprecipitated with antibodies specific for Histone H2AZ (Active motif # 39113) and acetylated Histone H2AZ (Abcam #ab18262; same H2A.Zac antibody used for IHC that recognizes both H2A.Z isoform 1 and 2). Normal Rabbit IgG (Upstate #12–370) or Sheep IgG (Santa Cruz Biotechnology #sc-2717) controls were also included for each corresponding ChIP assay and no precipitation was observed by quantitative Real-Time PCR (qPCR) analysis^5^. In total 10 μg of antibody was used for each immunoprecipitation. Input samples were processed in parallel. The antibody/protein complexes were collected by Protein A/G PLUS agarose beads (Santa Cruz sc-2003) and washed several times. The immune complexes were eluted with 1% SDS and 0.1 M NaHCO3 and samples were treated with proteinase K for 1 h and DNA was purified by phenol/chloroform extraction, ethanol precipitation, and resuspended in 30 μl H_2_O.

In total 10 ng of ChIP-DNA was used for library preparations using the TruSeq ChIP Sample Prep Kit (Illumina ®) following manusfacter’s instructions. Next generation sequencing was performed in the Illumina HiSeq 2000 at USC Epigenome Center, Illumina Genome Analyzer II at Ramaciotti Centre for Genomics (UNSW) or Illumina HiSeq 2500 at the Australian Genome Research Facility (AGRF) (GEO accession number GSE76336).

ChIP-seq reads from our samples and also public data (see Supplementary Table 2 with GEO additional accession numbers) were mapped to the human genome (hg19) using Bowtie (version 1.0.1)^54^, allowing up to three mismatches. Non-uniquely alignable reads were excluded.

For H2AZ and H2AZac, broad peaks were called using PeakRanger (version 1.16)^55^, peaks from replicates were combined by taking overlapping regions. Peaks for RNA Pol II, AR, and other transcription factors were called using MACS2^56^ The irreproducibility discovery rate^57^ method was used to combine replicate peaks.

For differential binding analysis for H2AZac ChIP-seq, the csaw package was used with a window size of 200 bp and normalized using a trended bias^58^. Blacklisted regions as specified by ENCODE were marked for exclusion from window counts of reads. Significant differential H2AZac gain in LNCaP and VCaP was defined as FDR < 0.05 and logFC > 0, and differential H2AZac loss as FDR < 0.05 and logFC < 0.

GSEA of the genes associated to H2A.Zac differential peaks was performed against the Molecular Signatures Database version 5.2 (MSigDB) hallmark gene sets (H) Collection^59^. Enrichment was assessed by hypergeometric testing as implemented in the R stats package.

Ngs.plot was used to visualize ChIP-seq signal^60^. Promoter-focus heatmaps were centered on the TSS coordinates from RefSeq hg19. DNAseI midpoints from ENCODE LNCaP and PrEC data were used to center on the heatmaps with enhancer focus and AR ChIP-seq midpoint for the AR-enhancer regions.

To calculate the significance of overlap between H2A.Z, H2A.Zac or AR and ChromHMM states Genomic Association Tester (GAT) was implemented^61^.

### Chromatin-state discovery and characterization

ChromHMM^20^ was applied to the chromatin modification aligned reads to simultaneously partition the genome of LNCaP, VCaP, and PrEC cell lines into 9 chromatin states. The following chromatin marks were used to create the different chromatin states for LNCaP and PrEC: H3K4Me3, H3ac, H3K4Me1, H3K27Me3, H3K36Me and H3K27ac; for VCaP: H3K4Me3, H3K4Me1, H3K27Me3, H3K36Me (GSE14092) and H3K27ac (GSE55062). Redundant states were then collapsed into seven distinct states and manually annotated by comparison to the published ChromHMM model for HMEC cells^62^. The annotated states were: Active promoter, bivalent promoter, active enhancer, poised enhancer, polycomb, transcribed region, and unmarked region (GSE76337).

### NOMe-seq

Cells were trypsinized and centrifuged for 7 min at 160×*g*, then washed in ice-cold PBS and resuspended in 1 mL ice-cold Nuclei Buffer (10 mM Tris,pH 7.4, 10 mM NaCl, 3 mM MgCl_2_, 0.1 mM EDTA and 1% IGEPAL, plus protease inhibitors) per 5 × 10^6^ cells and incubated on ice for 10 min. To obtain the nuclei, cells were dounce-homogenized for 15 times and incubated on ice for about 45 min, until single nuclei were visualized under the microscope. Nuclei were recovered by centrifugation at 500×*g* for 5 min and washed in Nuclei Wash Buffer (10 mM Tris, pH 7.4, 10 mM NaCl, 3 mM MgCl_2_ and 0.1 mM EDTA containing protease inhibitors). Freshly prepared nuclei (2 × 105 cells) were resuspended in 1× M.CviPI reaction buffer (NEB), then treated for 15 min with 150 U of M.CviPI (NEB M0227B; 50,000 U mL^−1^) in 15 µL 10× reaction buffer, 45 µL 1 M sucrose and 0.75 µL SAM in a volume of 150 μL. Reactions were quenched by the addition of an equal volume of Stop Solution (20 mM Tris–HCl [pH 7.9], 600 mM NaCl, 1% SDS, 10 mM EDTA, 400 µg mL^−1^ Proteinase K) and incubated at 55 °C overnight. DNA was purified by phenol/chloroform extraction and ethanol precipitation. The resulting DNA was used to perform either whole genome bisulfite sequencing or amplicon sequencing.

### Whole genome bisulfite sequencing

In total 50 ng of genomic DNA was bisulfite converted using the EZ DNA Methylation Gold kit (Zymo Research, Cat: D5005) following the manufacturer’s protocol. Prior to library preparation all samples were routinely checked for GpC treatment efficiency by clonal bisulfite sequencing of a nucleosome-free region located at GRP78 gene promoter (see Supplementary Table 4 for primer sequences and genomic location, and in Supplementary Fig. 4c for results). The bisulfite converted DNA was then subjected to library preparation using the TruSeq DNA Methylation kit, Illumina, Cat: EGMK81312). In this “post bisulfite conversion” library preparation method, single stranded bisulfite converted DNA was randomly primed to synthesize DNA strands with a specific sequence tag followed by 3′ tagging and PCR amplification using the FailSafe PCR enzyme (Gene Target Solutions,Cat: FSE51100). The PCR was performed for 10 cycles followed by purification using Agencourt AMPure XP beads (Beckman Coulter, Cat: A63881). The purified library was eluted in a final volume of 20ul pure water. Quality of the library obtained was checked using the DNA High sensitivity chip on the Agilent Bioanalyzer. The library was quantified using Qubit and KAPA Biosystems Library quantification kit according to manufacturer’s instructions. A total of 12 pMol of multiplexed libraries were loaded on a 70 bp PE rapid run on Illumina HiSeq2500.

### NOMe-seq analyses

Raw read pairs were aligned to the human genome (hg19) using bwa-meth^63^, a bisulfite aware wrapper for the bwa-mem aligner^64^. Read pairs with identical strand, start and end positions were considered PCR duplicates and removed from downstream analysis using MarkDuplicates from the Picard toolset. BisSNP v0.82.2^65^ was then used to extract the methylation status of each WCG and GCH site in each sample, which was then transformed into a “bigTable” of counts of methylation and coverage. Nucleosome depleted regions (NDRs) were detected using the “findNDRs” function of the aaRon R package https://github.com/astatham/aaRon. NDRs were detected by a 100 bp sliding window in 20 bp increments chi-squared test of GCH methylation vs. the genome background with a *p-*value cutoff of -log10(p) > 15, significant windows overlapped and retained if a minimum 140 bp in size. To enable the visualization and comparison of NOMe-seq profiles across sets of genomic regions (for example transcription start sites or transcription factor binding sites) the “methylationPlotRegions” family of functions was implemented in *aaRon*. The significance of differential chromatin accessibility and/or DNA methylation between cell lines or DHT conditions was calculated using t- test taking values around the center-point ± 5 bp of the NOMe plots. GEO accession number GSE76334.

### Targeted NOMe-seq

A total of 1 μg of genomic DNA was bisulfite treated, as previously described^66^. Bisulfite converted DNA was analyzed by bisulfite PCR analysis, as previously described^67^, using the primers and conditions listed Supplementary Table 3. The triplicate PCR products were pooled, cleaned up with the Wizard® SV Gel and PCR Clean-up System (Promega, Cat#A9282) and quantified. Then libraries for next-generation sequencing were prepared from these PCR products using the Illumina’s TruSeq DNA PCR-free Sample Prep kit (cat # FC-121-3001) following manufacturer’s instructions. The libraries were multiplexed and examined on the Agilent Bioanalyzer, and then quantified using the Kapa Biosystems Library Quantification kit according to manufacturer’s instructions. Then the libraries were pooled and a total amount of 10 pM was loaded into the MiSeq Desktop Sequencer (Illumina).

Paired-end fastq files were obtained for each library and aligned to hg19 using bwa-meth^63^. Downstream analysis was performed using the “ampliconAnalysis” function of the R package aaRon (http://github.com/astatham/aaRon). Data quality was checked by assessing the number of reads obtained and the bisulfite conversion efficiency per amplicon and per sample. All samples and amplicons had >×1000 coverage and high bisulfite conversion efficiency >98%. Percent methylation at each GpC site was calculated and used in all subsequent analysis.

For the validation of this new method, clonal bisulfite sequencing was performed on the same regions, as previously described^29^ (see also Supplementary Fig. 4c, d).

### RNA isolation and real time qPCR

Total RNA extraction was performed using Trizol Reagent (Invitrogen) following manufacturer’s instructions from LNCaP and 3R cells (±Dox) with or without DHT stimulation (0, 16 h) in biological triplicates or duplicates, respectively. The extracted RNA was treated with DNAseI (New England Biolabs) and cDNA synthesis was performed using SuperScript III Reverse Transcriptase (Invitrogen) and Random Hexamers (Invitrogen) following manufacturer’s instructions.

Enhancer RNAs primers were design, as previously described^68^ and are listed in Supplementary Table 4. Regions around 200–1,000 bp from the center of the AR binding site on the sense or anti-sense strand were selected and GRO-seq data (GSE63202) was used for more accurate primer design. In the case that AR enhancer was present inside a gene, primers were only design in the opposite strand of the gene to avoid accounting the mRNA expression. Real Time qPCR of the enhancer RNAs was performed, as previously described^5^.

### Gene expression arrays

Total RNA was extracted as above for LNCaP with or without DHT stimulation (0, 2 h, 4 h, 16 h) in biological triplicates for each of the timepoints. Affymetrix Human Gene 2.1 ST arrays were performed at Ramaciotti Centre for Genomics (UNSW). The efficiency of each DHT independent treatment was assessed prior array experiments by RT-qPCR of the androgen dependent genes KLK2 as previously described^5^ (Supplementary Fig. 6d). GEO accession number GSE76335.

The array data were preprocessed using the rma function (default parameters) in the R package oligo^69^. Differential expression between time points was assessed using the limma package^70^. To determine the number of gene significantly up-regulated and down-regulated, a log fold change of >/<0, respectively and a FDR <0.05 was used.

### GROseq data processing

GRO-seq fastq files from GSE63202 and GSE84432 were downloaded and aligned to hg19 using bowtie2 (version 2.2.4). The androgen-dependent eRNAs were selected from AR chromHMM-defined enhancers and centered to AR peaks and then split according to H2A.Zac presence.

### Data availability

All NGS and array data that support the findings of this study have been deposited in Gene Expression Omnibus (GEO) with the following accession numbers: Superseries: GSE76337; NoMe-seq: GSE76334; Gene expression arrays: GSE76335; ChIP-seq and ChromHMM: GSE76336.

In addition, public NGS data sets were analyzed in this study with the following accession numbers: GSE38685, GSE14092, GSE73785, GSE27823, GSE51621, GSE55062, GSE40050, GSE38391, GSE43791, GSE58428, GSE28264, GSE29692, GSE32970, GSE66729, GSE63202, GSE84432

Please see Supplementary Tables 2 and 3 for further information on the data sets.

The information of the clinical data set generated during and/or analyzed during the current study is available from the corresponding authors on reasonable request and under our Ethical Guidelines. qPCR raw files are available from the corresponding authors on reasonable request.

## Electronic supplementary material

Supplementary Information
