# Supplementary material for: Acetylated histone variant H2A.Z is involved in the activation of neo-enhancers in prostate cancer
Source: Nat Commun. 2017 Nov 7;8:1346. doi: 10.1038/s41467-017-01393-8 (PMC5676741; doi:10.1038/s41467-017-01393-8)
Supplement: Supplementary file 1 — Supplementary Information [file 41467_2017_1393_MOESM1_ESM.pdf]

Supplementary Information

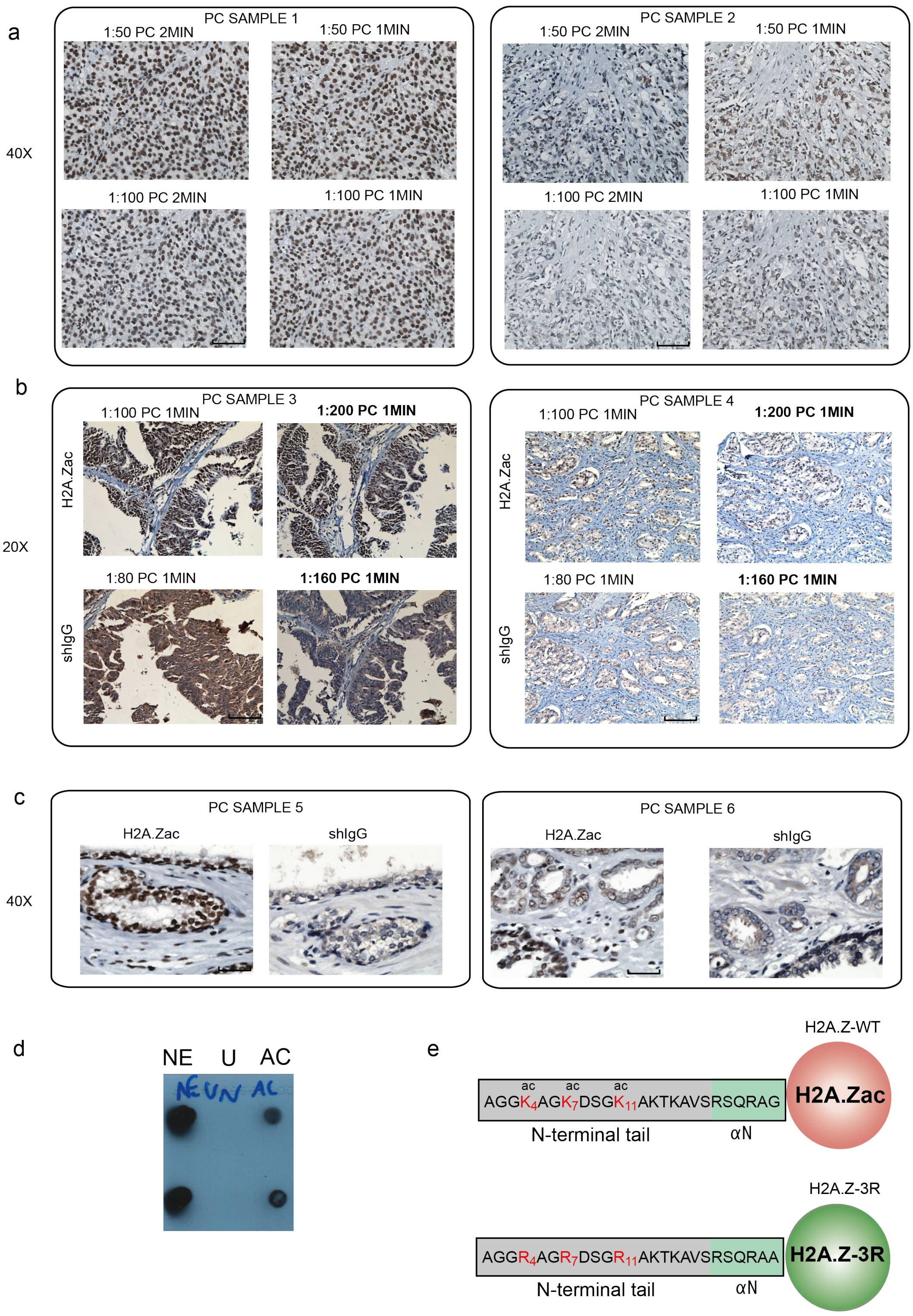

Supplementary Figure 1: H2A.Zac antibody characterisation and validation. (a) Two representative examples of H2A.Zac IHC in prostate cancer samples (PC) using different antigen retrieval conditions (pc: pressure cooker, 2 min or 1 min) and serial H2A.Zac antibody dilutions (1:50 and 1:100). The pictures were taken at 40X

magnification. Scale bar is 50μm **(b)** Additional two representative examples H2A.Zac IHC in prostate cancer samples (PC) using serial H2A.Zac antibody dilutions (1:100 and 1:200). Sheep IgG (shIgG) was used as negative control and the dilutions were adjusted to the same concentration to H2A.Zac condition. The chosen condition for IHC is highlighted in bold. The pictures were taken at 20X magnification. Scale bar is 100μm. **(c)** Two representative examples of the variable levels of expression of H2A.Zac in prostate cancer samples at the chosen condition and compared to shIgG negative control. The pictures were taken at 20X magnification. Scale bar is 100μm. The negative control showed some cytoplasmic background signal, however H2A.Zac was only present and therefore scored at the cell nuclei. **(d)** Dot Blot analysis to confirm the specificity of H2A.Z acetylation antibody (K4+K7+K11, Abcam #ab18262, 1:500 dilution factor) (N=4). MEL cells nuclear extract (NE), a recombinant peptide corresponding to the first 23 amino acids of the N-terminus of histone H2A.Z (AGGKAGKDSGKAKAKAVSRSQRA, Chinapeptides.Co.,Ltd. (**U**)) and a recombinant peptide corresponding to the first 23 amino acids of the N-terminus of histone H2A.Z that contain 3 acetylated lysines (K4, K7 and K11) (AGGK(Ac)AGK(Ac)DSGK(Ac)AKAKAV, Chinapeptides.Co.,Ltd. (**AC**),). **(e)** Schematic representation of the three mutations incorporated in H2A.Z gene construct at the three lysines most commonly acetylated in human H2A.Z (highlighted in red, K4, K7 and K11). These three lysines were replaced with arginines (shown as H2A.Z-3R) to act as a dominant negative H2A.Zac model. Acetylated H2A.Z wild type sequenced is also shown (H2A.Z-WT) as comparison.

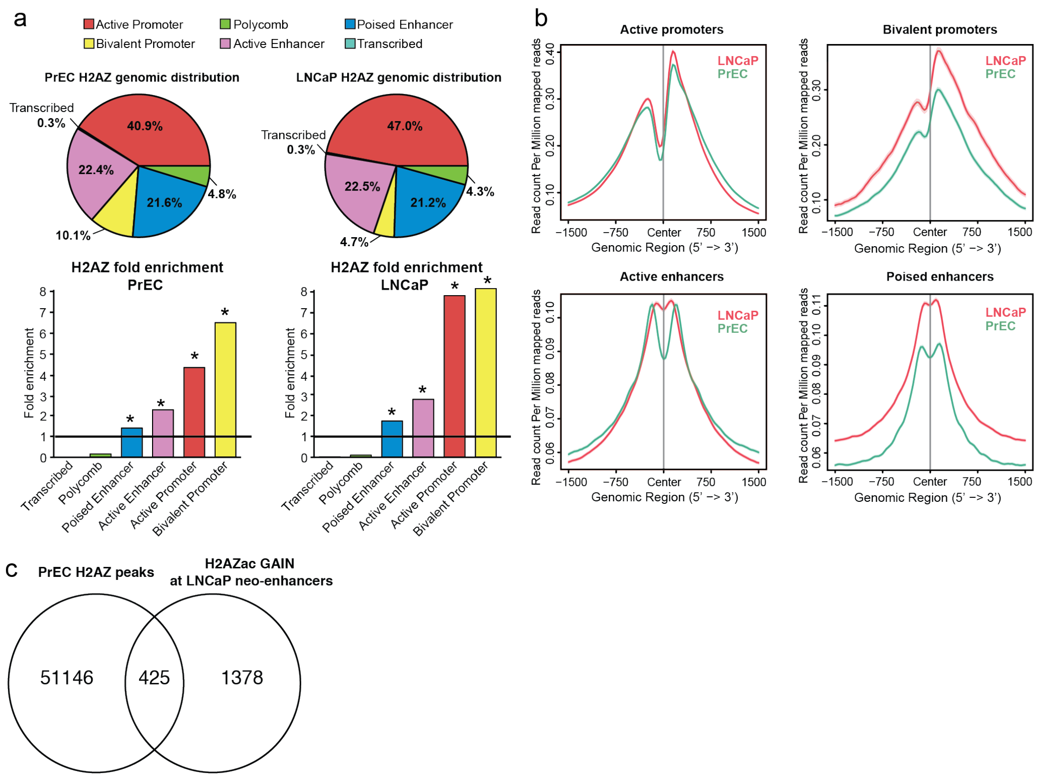

Supplementary Figure 2: Total H2A.Z occupancy at genomic regions in PrEC and LNCaP. (a) GAT of H2A.Z ChIP-seq and ChromHMM regions in LNCaP and PrEC cells. A total number of 51,650 H2A.Z intersecting peaks from biological replicates were detected in PrEC and 46,906 H2A.Z intersecting peaks in LNCaP. Pie charts (upper panel) representing the percentage of marked H2A.Z peaks falling in each ChromHMM state. Observed versus expected fold enrichment graphs (lower panel), * *p value* <0.0001 of significant enrichment. The line indicates the threshold of the significant enrichment. **(b)** Ngs.plot of the average signal (read count per million mapped reads) of H2A.Z ChIP seq in LNCaP (red) and PrEC (green) cells in each genomic regions of significant enrichment. The plots were centred at the transcriptional start site (TSS) in the case of active and bivalent promoters and to the midpoints of DNase I hypersensitive sites (DNAseI) peaks in the case of active or poised enhancers. **(c)** Venn diagrams showing the overlap between all H2A.Z peaks called in PrEC and H2A.Zac peaks gained at active enhancer in LNCaP.

a

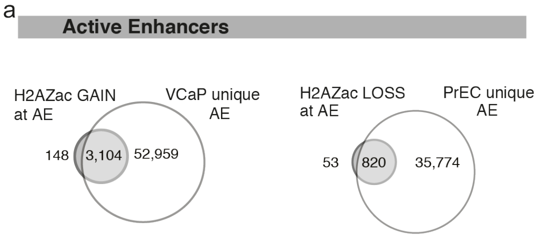

b

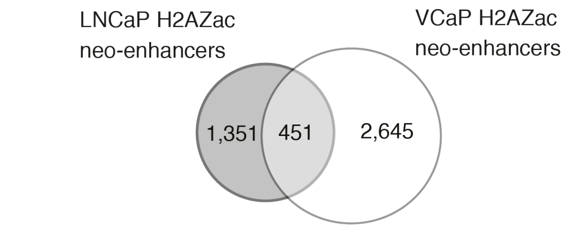

c

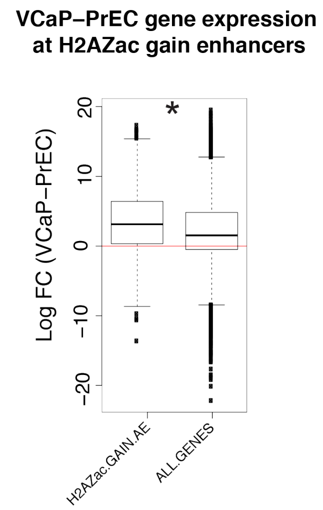

d

GSEA at LNCaP H2A.Zac GAIN neo-enhancers

Term	Fold Enrichment	p value
HALLMARK_ANDROGEN_RESPONSE	2.29	9.06E-04
HALLMARK_OXIDATIVE_PHOSPHORYLATION	0.34	2.23E-03
HALLMARK_G2M_CHECKPOINT	0.34	2.50E-03
HALLMARK_KRAS_SIGNALING_DN	1.93	2.93E-03
HALLMARK_ESTROGEN_RESPONSE_EARLY	1.69	7.12E-03
HALLMARK_UNFOLDED_PROTEIN_RESPONSE	0.24	8.89E-03
HALLMARK_DNA_REPAIR	0.36	1.13E-02
HALLMARK_E2F_TARGETS	0.48	1.69E-02
HALLMARK_XENOBIOTIC_METABOLISM	1.58	2.66E-02
HALLMARK_MYOGENESIS	1.57	3.14E-02
HALLMARK_MYC_TARGETS_V1	0.55	3.52E-02
HALLMARK_ESTROGEN_RESPONSE_LATE	1.48	4.20E-02

GSEA at VCaP H2A.Zac GAIN neo-enhancers

Term	Fold Enrichment	p value
HALLMARK_ESTROGEN_RESPONSE_EARLY	2.06	1.40E-06
HALLMARK_ESTROGEN_RESPONSE_LATE	1.93	1.82E-05
HALLMARK_MYOGENESIS	1.96	1.89E-05
HALLMARK_E2F_TARGETS	0.32	1.09E-04
HALLMARK_MYC_TARGETS_V1	0.32	1.09E-04
HALLMARK_APOPTOSIS	0.52	1.70E-02
HALLMARK_ANDROGEN_RESPONSE	1.56	4.07E-02
HALLMARK_KRAS_SIGNALING_DN	1.40	4.08E-02
HALLMARK_SPERMATOGENESIS	0.54	4.38E-02
HALLMARK_G2M_CHECKPOINT	0.65	4.76E-02

e

GSEA at LNCaP H2A.Zac LOSS enhancers

Term	Fold Enrichment	p value
HALLMARK_MYOGENESIS	2.07	3.85E-05
HALLMARK_MYC_TARGETS_V1	0.27	8.46E-05
HALLMARK_UV_RESPONSE_DN	2.02	2.51E-04
HALLMARK_P53_PATHWAY	1.66	2.70E-03
HALLMARK_TNFA_SIGNALING_VIA_NFKB	1.68	2.77E-03
HALLMARK_PI3K_AKT_MTOR_SIGNALING	0.22	3.76E-03
HALLMARK_SPERMATOGENESIS	0.22	4.08E-03
HALLMARK_KRAS_SIGNALING_DN	1.74	5.28E-03
HALLMARK_FATTY_ACID_METABOLISM	0.43	1.11E-02
HALLMARK_TGF_BETA_SIGNALING	2.02	2.29E-02
HALLMARK_HEME_METABOLISM	0.55	2.58E-02
HALLMARK_KRAS_SIGNALING_UP	1.47	3.42E-02
HALLMARK_EPITHELIAL_MESENCHYMAL_ TRANSITION	1.41	4.54E-02

GSEA at VCaP H2A.Zac LOSS enhancers

Term	Fold Enrichment	p value
HALLMARK_TNFA_SIGNALING_VIA_NFKB	2.51	1.54E-04
HALLMARK_KRAS_SIGNALING_DN	2.34	1.14E-03
HALLMARK_MYOGENESIS	1.98	1.03E-02
HALLMARK_INFLAMMATORY_RESPONSE	1.84	2.32E-02
HALLMARK_MYC_TARGETS_V1	0.39	4.59E-02
HALLMARK_UV_RESPONSE_DN	1.81	4.84E-02
HALLMARK_HEME_METABOLISM	0.40	5.40E-02
HALLMARK_FATTY_ACID_METABOLISM	0.35	6.48E-02
HALLMARK_UNFOLDED_PROTEIN_RESPONSE	1.85	6.70E-02
HALLMARK_APICAL_SURFACE	2.37	8.65E-02
HALLMARK_PI3K_AKT_MTOR_SIGNALING	0.25	8.82E-02
HALLMARK_UV_RESPONSE_UP	0.50	1.42E-01
HALLMARK_ALLOGRAFT_REJECTION	0.57	1.63E-01
HALLMARK_BILE_ACID_METABOLISM	0.47	1.98E-01
HALLMARK_OXIDATIVE_PHOSPHORYLATION	0.65	2.11E-01

Supplementary Figure 3: Characterisation of cancer neo-enhancers in the androgen-sensitive prostate cancer cell line VCaP. (**a**) Venn diagrams overlapping the H2A.Zac gained (LHS) or lost peaks (RHS) at VCaP active enhancers with the corresponding "unique" active enhancers. The terminology "unique" was used to call enhancers that were only present in VCaP but not in PrEC, for the gained peaks; and the enhancers that were only present in PrEC but not in VCaP for the lost peaks. (**b**) Venn diagrams to overlap H2A.Zac neo-enhancers in LNCaP and VCaP. **(c)** Box plots for the logarithmic fold change expression (logFC) of VCaP minus PrEC to compare differential gene expression between all genes and genes associated with H2A.Zac neo-enhancers in VCaP from (a). To associate gene expression with enhancers, all the genes upstream or downstream of a particular enhancer within a 25kb window were assigned as an enhancer-gene association. * t-test *p-value*<0.0001. (**d**) Gene set Enrichment Analyses (GSEA) of the genes associated with unique active enhancers (neo-enhancers) with gain of H2A.Zac in LNCaP (n=1,803) (LHS) in VCaP cells (n=3,104) (RHS). Hypergeometric test on MSigDB gene-sets was performed using all genes within 25KB of those enhancer regions. The tables show fold enrichment and p-value of the significant gene sets (p-value $\leq$ 0.05) from the Hallmark collection. The pathways highlighted in blue are common between cell lines (**e**) GSEA of the genes associated with loss of H2A.Zac in LNCaP (n=1,932) (LHS) in VCaP cells (n=820) (RHS) at PrEC unique active enhancers. Hypergeometric test on MSigDB gene-sets was performed using all genes within 25KB of those regions. The tables show fold enrichment and p-value of the significant gene sets (p-value $\leq$ 0.05) from the Hallmark collection.

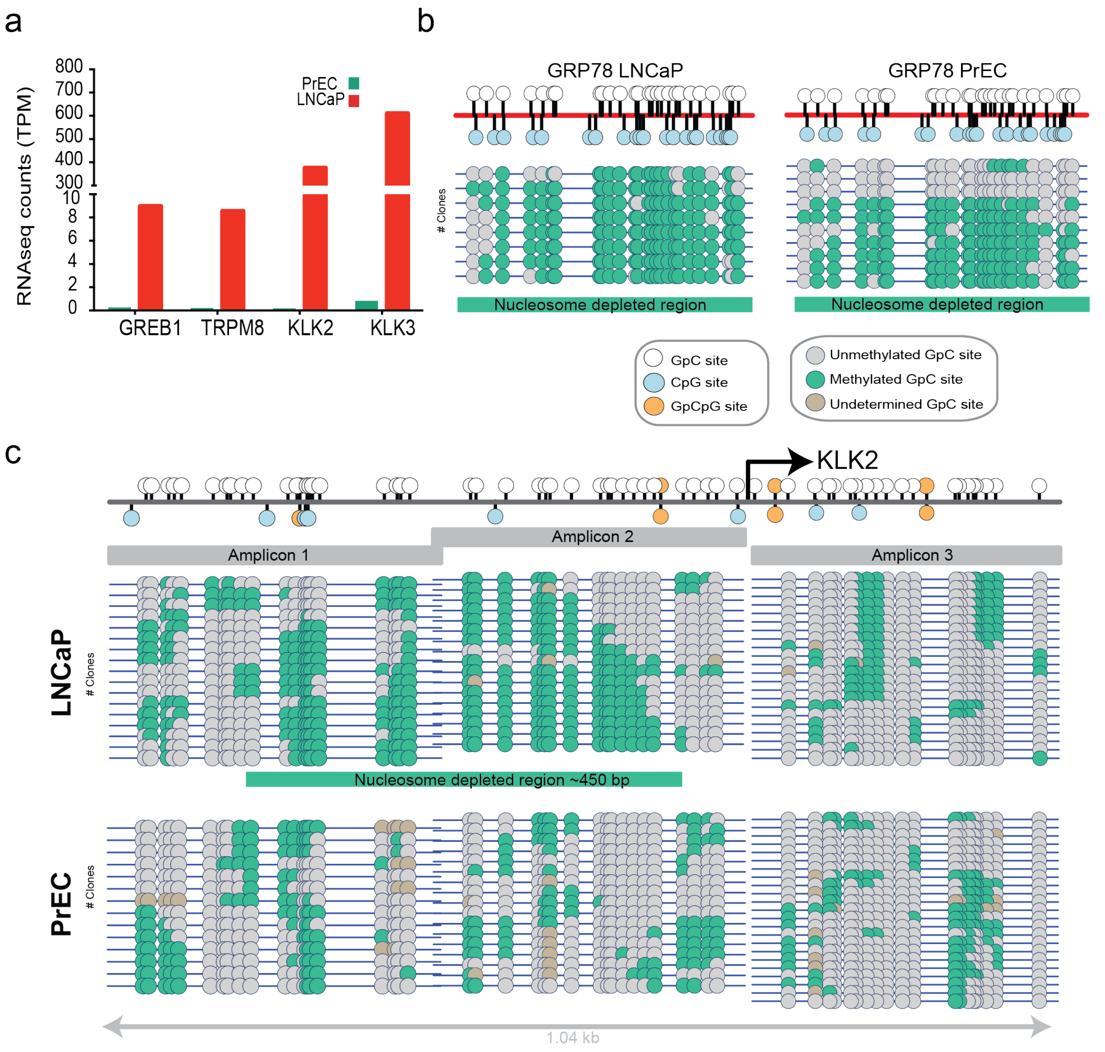

Supplementary Figure 4: Expression and chromatin accessibility validation for redistributed H2A.Zac at promoters and/or enhancers in cancer. (a) RNA seq data from PrEC (green) and LNCaP (red) represented as Transcripts Per Million (TPM) for four gene examples. **(b)** NOMe validation by bisulphite clonal sequencing at accessible and occupied regions. GRP78 gene promoter was used as positive control for accessibility to test the efficiency of M.CviPI GpC methyltransferase transferase. The Figure shows an outline of the region PCR cloned where the blue circles correspond to CpG sites and the white circles represent GpC site. Underneath of this map each sequenced clone is represented and the GpC methtylation status of each one is colour coded, methylated GpC sites are the green circles and the unmethylated GpC are represented as grey circles. A green rectangle spans the presence of a nucleosome-depleted region (NDR) when applicable. **(c)** NOMe validation by bisulphite clonal sequencing of KLK2 promoter was used as the gene control for the validation of the targeted NOMe-seq technique. Three PCRs were designed around the TSS of KLK2 gene and the NOMe clonal sequencing was performed in both cell lines. An outline of the area of KLK2 promoter analysed is shown and GpC sites are highlighted in white circles, CpG sites in blue circles and the GpCpG sites, which had to be ruled out for the GpC analysis, are highlighted in orange circles. Below the map, each sequenced clone is represented and the GpC methtylation status of each one is colour coded, methylated GpC sites are the green

circles and the unmethylated GpC are represented as grey circles. The methylation status of the GpC sites that couldn't be determined by Sanger sequencing is shown as brown circles. This data was used to compare them with the targeted NOMe-seq results to validate this method.

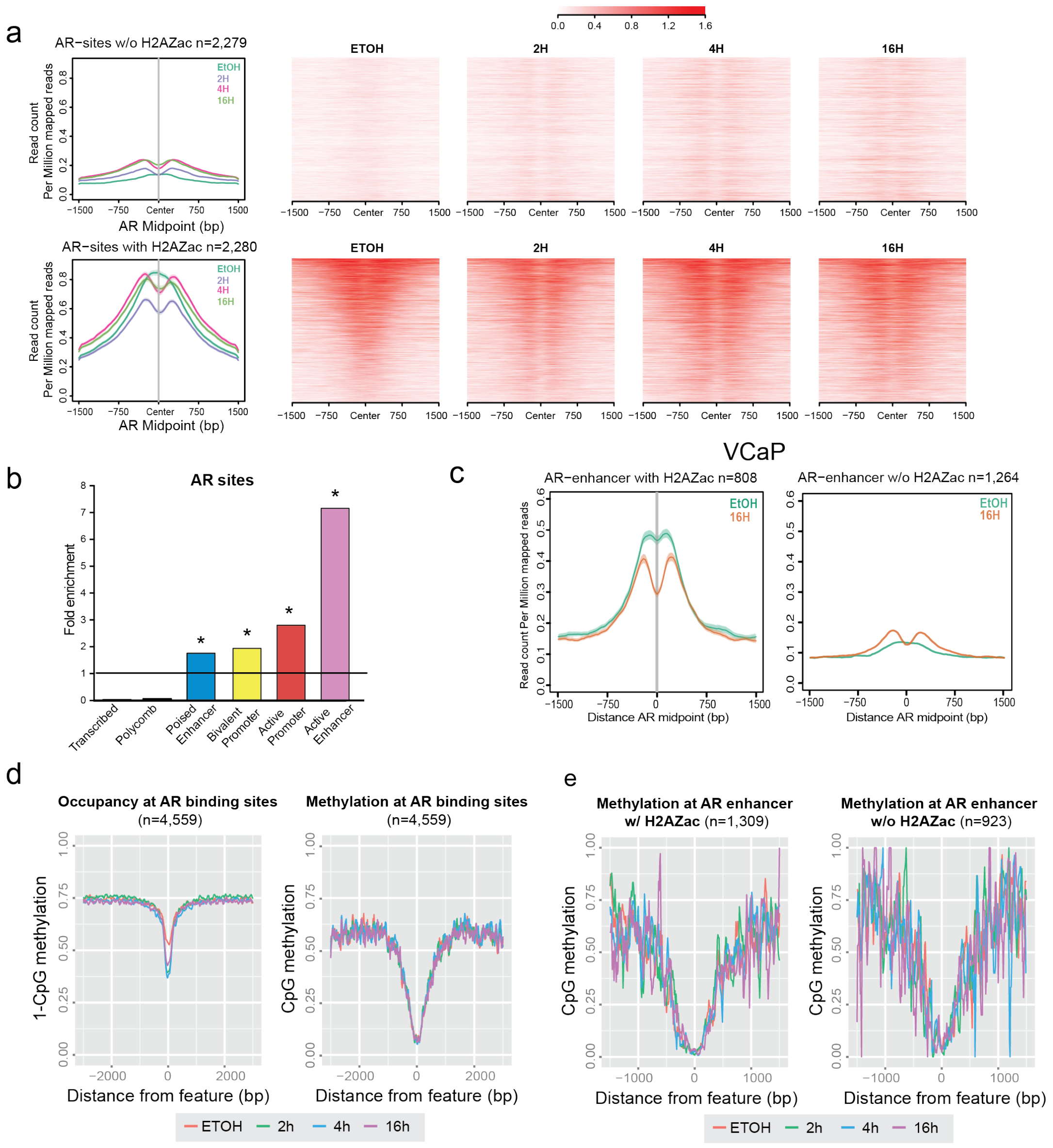

Supplementary Figure 5: Complementary analysis of H2A.Zac at all AR binding sites during DHT treatments. (a) LHS, H2A.Zac ChIP-seq average intensity profile using ngs.plot during a time course of DHT treatment, from the androgen-deprivation conditions (EtOH), to the following DHT timepoints: 2h, 4h and 16h. All AR sites were split according to the presence of H2A.Zac. 50% of the AR sites (n=2,279) have none or very low levels of H2A.Zac (upper plot) and the other 50% (n=2,280) present H2A.Zac in a distinct intensity and distribution throughout the time course (bottom plot). At the RHS the same ngs.plot were represented as heatmaps using the same scale in all cases. Scale bar shows the colorkey of the intensity of H2A.Zac ChIP average signal (read count per million mapped reads) **(b)** Observed versus expected fold enrichment GAT graphs of AR ChIP-seq and ChromHMM regions in LNCaP cells. A total number of 4,559 of AR intersecting peaks from biological replicates were detected in LNCaP cells treated with 10nM of DHT for 4h (GSE40050). The ChromHMM used correspond to LNCaP growing in normal media, in which AR is active and bound to the DNA. * *p-value* <0.0001 of significant enrichment. The line indicates the threshold of the significant enrichment. AR is mostly enriched at active

enhancers. **(c)** H2A.Zac ChIP-seq average intensity profile on the centered AR peaks using ngs.plot in VCaP cells under androgen-deprivation conditions (EtOH, green) or after 16h of DHT treatment (DHT, orange). AR active enhancers sites in VCaP (n=GSE55062) were split according to the presence of H2A.Zac. 40% of the AR enhancer sites (n=808) had H2A.Zac presence (LHS) and the other 60% of the AR enhancer sites (n=1,264) have none or very low levels of H2A.Zac (RHS). **(d)** NOMe plots representing chromatin accessibility (LHS) and DNA methylation (RHS) at all AR sites (n=4,559). Chromatin accessibility was determined by NOMe-seq at the control (EtOH, red) and the timepoints of DHT treatment (2h, green; 4h, blue and 16h, purple) and it is represented as 1-methylated GpC ratio and CpG methylation ratio, respectively. **(e)** NOMe plots for DNA methylation comparison among the different DHT conditions (EtOH, red; 2h, green; 4h, blue and 16h, purple) at the split groups of AR enhancer with H2A.Zac (w/H2A.Zac, n= 1,309) and without H2A.Zac (w/o H2A.Zac, n= 923).

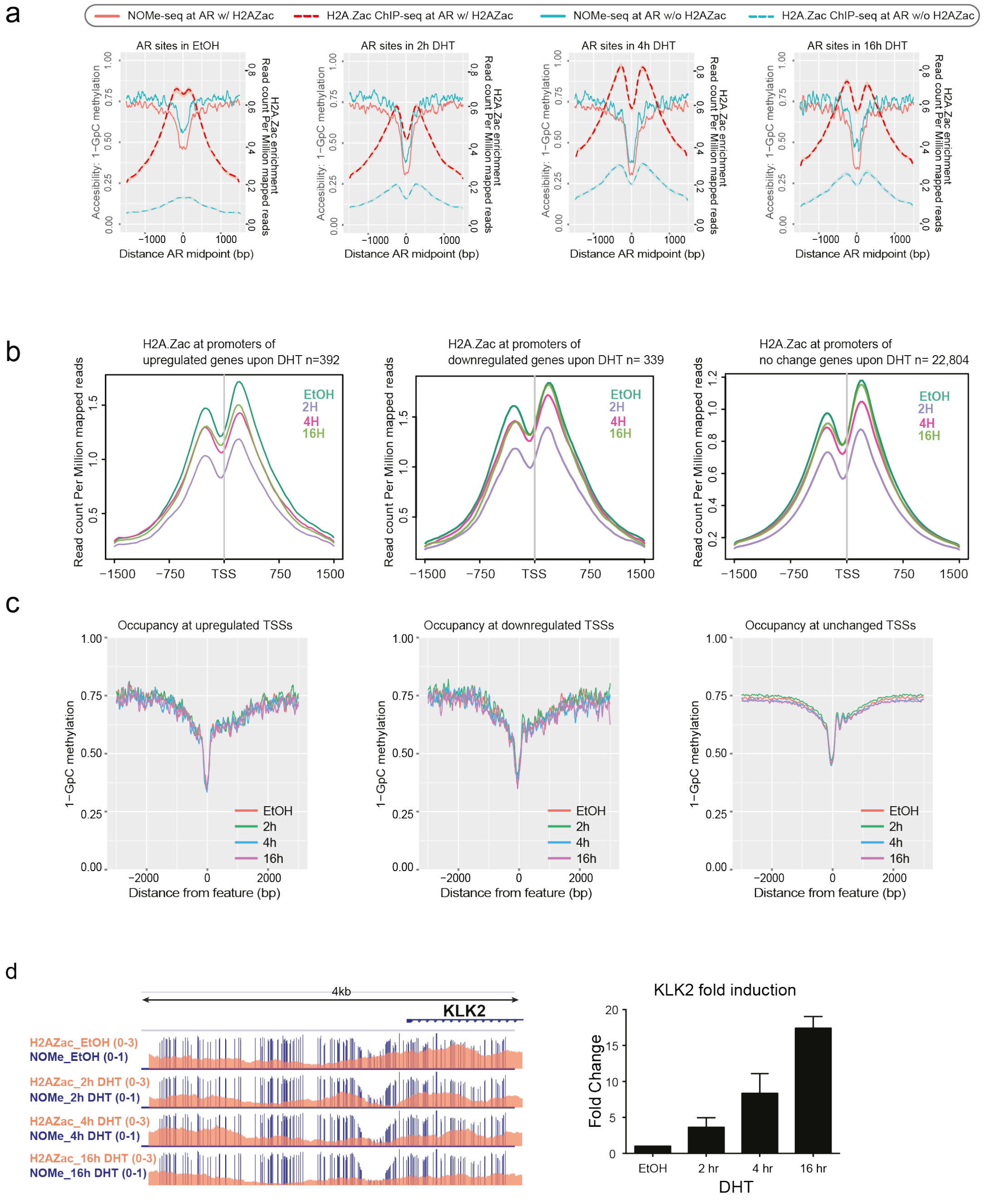

Supplementary Figure 6: **Chromatin accessibility and H2A.Zac dynamics at AR enhancers and promoters of androgen-dependent genes**. **(a)** Merged NOMe plots (full lines) with H2A.Zac ChIP-seq average signal (dashed lines). Each plot represents the data for one timepoint of the AR enhancers with H2A.Zac (red) and w/o H2A.Zac (blue). **(b)** Ngs.plot of H2A.Zac ChIP-seq average signal of the DHT time course at the gene promoters of up-regulated (n=392, LHS plot), downregulated (n=339, middle plot) and unchanged (n=22,804, RHS plot) genes upon 16h of DHT treatment. **(c)** NOMe plots for chromatin accessibility (represented as the ratio 1-mGpC) of the promoters of the same genes from (b) across DHT timepoints. **(d)** LHS, merged IGV tracks of control (EtOH) and DHT timepoints (2h, 4h and 16h) showing H2A.Zac ChIP-seq signal (orange) overlapped with targeted NOMe-seq, represented as 0-1

ratio of each 1-mGpC site (dark blue bars), of the promoter of the androgen-sensitive gene KLK2. RHS, RT-qPCR for KLK2 expression during DHT treatment for biological triplicates, represented as Fold change with respect to the control, EtOH. All error bars are shown as s.d.

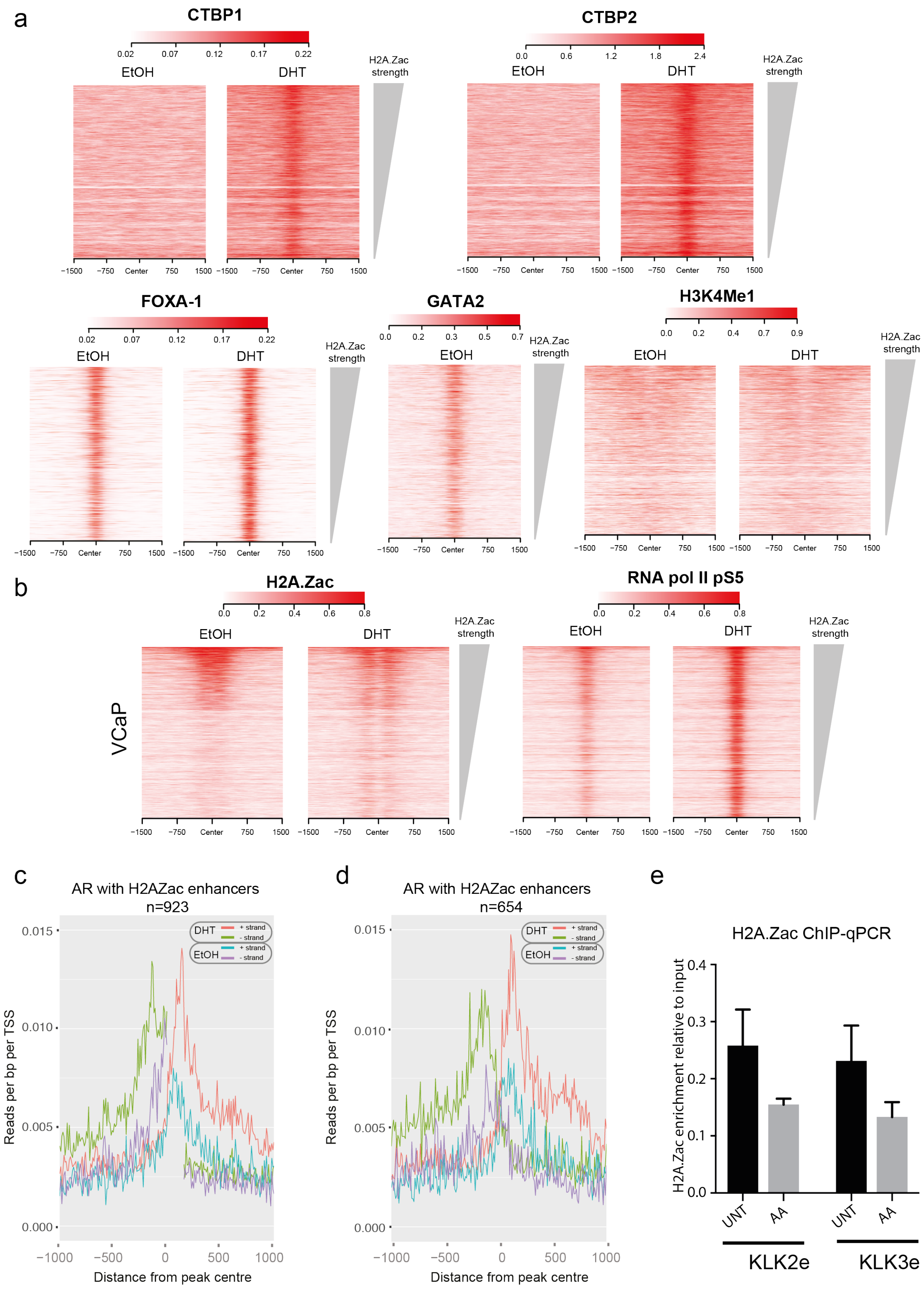

Supplementary Figure 7: **AR enhancers flanked by H2A.Zac-nucleosomes have features of functionally active enhancers**. **(a)** Heatmap of CTBP1, CTBP2, FOXA-1, GATA-2 and H3K4Me1 ChIP-seq data at AR enhancers (n=2,232) ordered by H2A.Zac signal intensity from highest (top) to lowest (bottom) at starving conditions (EtOH) and DHT treatment for (DHT) in LNCaP cells. Scale bars show the colorkey of the intensity of each corresponding ChIP average signal **(b)** Heatmap of H2A.Zac (LHS) and RNApolII

pS5 (RHS) ChIP-seq data at AR enhancers (n=2,072) ordered by H2A.Zac signal intensity from highest (top) to lowest (bottom) at starving conditions (EtOH) and DHT treatment for (DHT) in VCaP cells. Scale bars show the colorkey of the intensity of each corresponding ChIP average signal. **(c) and (d)** Bidirectional histograms (+ and – strands) of eRNA profile from GROseq data around AR enhancers with H2AZac during starvation (EtOH) and after 1h of DHT treatment (DHT) in LNCaP cells. The number of eRNAs were downsampled as quality check in order to balance the number of eRNA compared between AR with H2A.Zac or without H2A.Zac. **(c)** shows shuffled and randomly peak 923 regions and **(d)** shows 654 regions proportionally located in the genome. **(e)** H2A.Zac ChIP qPCR at AR enhancers of KLK2 enhancer RNA (KLK2e) and KLK3 enhancer RNA (KLK3e) in LNCaP cells either untreated (UNT) or treated (AA) with Anacardic Acid at 90µM for 48h. Data is presented as the average of three independent experiments normalised with the input. All error bars are shown as s.d.

Supplementary Table 1: Clinical features of the prostate cancer cohort

	N	mean
Age at RP	63	62.21±6.16
Preoperative PSA (ng/ml)	63	9.91± 6.43
	N	% of H2A.Zac positive cells
Biochemical relapse		
Yes	22	34.92
No	41	65.08
Margin status		
Negative	39	61.90
Positive	24	38.09
Extraprostatic extension		
Yes	30	47.62
No	33	52.38
Gleason score		
< 6	15	23.81
7	34	53.97
> 8	14	22.22
Stage		
< 2C	32	50.79
> 3A	31	49.21

Supplementary Table 2: ChIP-seq Datasets used in this publication

Target	Cell Line/s	Source	Company and Cat # ChIP-seq validated Abs	Accession
H2A.Z	PrEC/LNCaP	This publication	Active Motif Cat #39113	GSE76336
H2A.Zac	PrEC/LNCaP/ LNCaP +/- DHT/VCaP/V CaP +/- DHT/	This publication	Abcam ab18262	GSE76336
H3K4me3	PrEC/LNCaP	Bert et al., Cancer Cell 2013	Abcam ab8580	GSE38685
	VCaP	Ju et al., Cancer Cell 2010		GSE14092
H3K27me3	PrEC/LNCaP	Bert et al., Cancer Cell 2013	Millipore #07-449	GSE38685
	VCaP	Ju et al., Cancer Cell 2010		GSE14092
H3K4me1	PrEC	This publication	Active Motif Cat #39297	GSE76336
	LNCaP	Taberlay et al., Genome Res 2016	Active Motif Cat # 39298	GSE73785
	LNCaP +/- DHT	Wang et al., Nature 2011	Abcam ab8895	GSE27823
	VCaP	Ju et al., Cancer Cell 2010		GSE14092
H3K27ac	PrEC	This publication	Active Motif Cat #39133	GSE76336
	LNCaP	Taberlay et al., Genome Res 2016		GSE73785
	LNCaP +/- DHT	Hazelett et al., PLoS Genet 2014		GSE51621
	VCaP	Asangani et al., Nature 2014	Abcam ab4729	GSE55062
H3K36me3	PrEC/LNCaP	This publication	Abcam ab9050	GSE76336
	VCaP	Ju et al., Cancer Cell 2010		GSE14092
H3ac	PrEC/LNCaP	This publication	Millipore #06-599	GSE76336
AR	LNCaP +/- DHT	Decker et al., Nucleic Acids Res. 2012	Abcam ab74272	GSE40050
	VCaP +/- DHT	Asangani et al., Nature 2014	Millipore #06-680	GSE55062
GATA2	LNCaP + DHT	BioProject	Santa Cruz Biotech sc-9008	GSE38391
FOXA1	LNCaP + /- DHT	Chen et al., EMBO J 2015	Abcam ab23738	GSE43791
CTBP1	LNCaP + /- DHT	Takayama et al., Cancer Res 2014	BD biosciences Cat #612942 *	GSE58428
CTBP2	LNCaP + /- DHT	Takayama et al., Cancer Res 2014	Abcam, ab96107 *	GSE58428
Pol II phospho S5	LNCaP + /- DHT	Tan et al., Mol Cell Biol 2012	Abcam, ab5131	GSE28264
	VCaP +/- DHT	Asangani et al., Nature 2014	Abcam, ab5408	GSE55062

*Abs not validated for ChIP-seq elsewhere

Supplementary Table 3: Other Datasets used in this publication

Data Type	Cell Line/s	Source	Accession
NOMe-seq	LNCaP/PrEC/ LNCaP +/- DHT	This publication	GSE76334
DNAseI-seq	PrEC	ENCODE	GSE29692
	LNCaP	ENCODE	GSE32970
RNA-seq	PrEC/LNCaP	Taberlay et al., Genome Res 2016	GSE73785
	VCaP	Balbin et al., Genome Res 2015	GSE66729
Affy expression arrays	LNCaP +/- DHT	This publication	GSE76337
GRO-seq	LNCaP +/- DHT	Puc et al., Cell 2015	GSE63202
	VCaP +/- DHT	Toropainen et al., Sci Rep 2016	GSE84432
ChromHMM	PrEC	This publication	GSE76336
	LNCaP	This publication	GSE76336
	VCaP	This publication	GSE76336

Supplementary Table 4. Sequence and genomic localisation of primers used in this study

Amplicon name	Forward Primer	Reverse Primer	Genome localisation (hg19)	Amplicon size (bp)
NOMe primers				
GREB1 ENHANCER REGION				
GREB1_1	TTTTTATGATAGGGTTTTATTTTGTTATTT	ACAACAAAAACCCCCTAAAAAA	chr2: 11679192 - 11679531	340
GREB1_2	TGAAGATTTTTTGGGYTTTTTT	CCTAAAATATTTTRCTAAATCACAA	chr2: 11679494 - 11679832	339
GREB1_3	TGTGGYTTTAGTTTAAGTATATAAATTTTT	TAATCTATRCCTAAAAATAAAAAAAA	chr2: 11679708 - 11680125	418
GREB1_4	AGATATTAGYTTTGAAATTTTTTTTT	AAAAACACCCRTTCATTAAATAA	chr2: 11679968 - 11680413	446
GREB1_5	AGTTTATTTAATGAAYGGGTGTTT	ACAAAATATCAAAAAATATTTCTTTAAAA	chr2: 11680388 - 11680692	305
TRPM8 ENHANCER REGION				
TRPM8_6	GAATTTTGGGATTTTTATTTTTTT	CCCTTAAATTTAAAAATTAAAAAAATA	chr2: 234861543-234861822	280
TRPM8_7	TAGATTTTGAGATTTYGTTTTTTATTT	AAAAAAAAAARCTTAAAAACTAAAA	chr2: 234861847-234862149	303
TRPM8_8	TAATATAGAAAATTTTTTATTTGTTTTTTT	CCAACAAATTTTAARCATAAAA	chr2: 234862099-234862532	434
TRPM8_9	ATATTTTATTTTTATTGTAGTTAAGGATTT	ACAAARCTAACACTTCAAAAAA	chr2: 234862390-234862701	312
TRPM8_10	GAAATTTTYGAATGTTTTTATTTATTAAT	CCACTATTAAAAATARCTCTTTAAAA	chr2: 234862567-234862935	369
KLK2 PROMOTER REGION				
KLK2_1	TATTTGTATTATATTTTGTAATAGGGATTATTT	AACCTAACCCTCCATTCTAACTAA	chr19: 51374155 - 51374407	253
KLK2_2	GTATGAAGATTAGTTAGAATGGAGGGTT	CTTAAATAAAAACCACCTAAATCCAATA	chr19: 51374375 - 51374820	446
KLK2_3	ATTGGATTTAGGTGGTTT	CCTAATATTAACTCTTATTATAAAAATCCACTA	chr19: 51374794 - 51375150	357
KLK2_4	TAGTGGATTTTTATAATAAGAGTTAATATTAGGATTT	TAAAAAATCACTTAAAATCAAAAATTCAAA	chr19: 51375118 - 51375479	362
KLK2_5	TTTGAATTTTTGATTTTAAGTGATTTTTT	CTTATAATACCACATATTATCCAATCCAA	chr19: 51375450 - 51375810	361
KLK2_6	TTTGGATTGGATAATATGTGGTATTAT	CTAATTTTTAATTTTTTTATAAAAAAAAAATCTA	chr19: 51375781 - 51376209	429
KLK2_7	TGTAATTTTATTATTTTGGGAGAT	CCTATAAATCATAAAAATATAATCTAAAA	chr19: 51376096 - 51376461	369
KLK2_8	TTTGTTTTTTAGATTATATTTTTATGATTT	TCCACARCCAAATAATAAATTTA	chr19: 51376427 - 51376716	290

KLK2_9	GTGATAGGGTTTA AGGAATTAGTGG	TCAACATATAAAA CCTAATTCTCTCCA T	chr19: 51376725 - 51377112	388
KLK2_10	TTTGYTTGAGTTGT TTGTTGYTTTG	TAARCTACCTCCTA AACCCCTC	chr19: 51377113 - 51377512	400
KLK2_11	GGGTTTTAAGAGA ATAAGAGGTTTTA GAAATT	CAAAAAACCCAAA TTCCACCAA	chr19: 51377227- 51377629	403
KLK2_12	GGATTAGGATTGG GAGTTTAGGATA	CCTTAATTTCTAAA ACCTCTTATTCTCT TAA	chr19: 51377598 - 51377882	285
POSITIVE CONTROL FOR NUCLEOSOME ACCESIBILITY				
GRP78	GAGAAGAAAAAGT TTAGATTTTATAG	AAACACCCCAATA AATCAATC	chr9: 128003577- 128004030	454
eRNAs primers				
KLK3_S2	AGAATTGCCTCCCA ACACTG	GGACCCACTCCTCA CTCAAA	chr19: 51354310- 51354554	245
KLK3_S3	GATGTGGGAATGG CCTAAGA	GGATTAGGTGGCA AGATGGA	chr19: 51354679- 51354901	223
KLK3_AS1	TTTTGTGAATGCTG GCAGAG	TGCACAGCATCCAC CTAGAC	chr19: 51353791- 51353952	162
KLK3_AS2	GACGATCAAATGT GGTCACG	CTCCATCAAATGAG GCCAGT	chr19:51353211- 51353398	188
KLK2_S1	TTGCTCAGAAGAC ACACAGAAACATA	GGCTCTGGGTGAA CAGTGTTG	chr19: 51373052- 51373122	71
KLK2_S2	GGTCCCAGCTTCCG AGGTA	GGATACGGGATTCT TAGACCTCTCA	chr19: 51373437- 51373497	61
KLK2_AS1	TGCTCACCTTTGCC ACAAAC	ATGGGATGATCAG AGCAGTTCA	chr19: 51372700- 51372758	59
KLK2_AS3	TATGATGGGCTCCT GGTATCATAGA	CTTTCCTTGACCCC CTATCTCA	chr19: 51372306- 51372383	78
ZBTB16_AS1	AGCAAATCCTGGC TGAAAGG	CCTTGAACCATATC CTGGACCTA	chr11: 114049829- 114049891	63
ZBTB16_AS2	CCAGAGTGGCTTG GAAAGCT	GTGTTTCTAAGCCT GTTGGATTACC	chr11: 114049277- 114049348	72
ZBTB16_AS3	TGGGCCTGTTTCTT ACCTAGAAA	TCAACCAACCCTGT GGCATA	chr11: 114049068- 114049127	60
GHRHR_AS1	GTTTCCGTGGCCAA GTGTTT	CCACAGGAGAGCT GGGATTG	chr7: 31010184- 31010302	119
GHRHR_AS2	TCATGCTTTACCTC TCAGAGATCAA	TGCCAGGCACTCCC AAAC	chr7: 31010058- 31010124	67
LRRC16A_A S1	TGCTTTGCACAAGT TCATCATTAA	AGTCTGGGCATCA GCTCATTATC	chr6: 25330118- 25330186	69
LRRC16A_A S2	CCCTGTTATCTTTT CATTGTTTTCTTC	ACTCAAAGTATCTC AGCTCTTGTTGTAT G	chr6: 25329973- 25330049	77
ChIP primers				
KLK2_enh_C hIP	AAGGAGGCAGCAT GAACTTTG	GCTCTGATCATCCC ATATGAATTG	chr19: 51372645- 51372715	71
KLK3_enh_C hIP	GCTGTCTTTGCTCA GAAGACACA	CAGTGTTGGGAGG CAATTCTC	chr19: 51354265- 51354329	65
H2A.Z-3R primers				
H2AZ_mut_i nt	CGTGGGCAGAATC CACA	GAGAGTCCAGTTCC TCGTCG	NA	211
